# Reconfigurable intelligent surface and UAV coordination for reliable THz wireless networks

**DOI:** 10.1371/journal.pone.0345290

**Published:** 2026-03-23

**Authors:** M. Rudra Kumar, Ravi Uyyala, M. Ramchander, Y. Ramadevi, Ramesh Babu Palamakula, E. Padmalatha, Deema Mohammed Alsekait, Diaa Salama AbdElminaam, Premkumar Chithaluru

**Affiliations:** 1 Department of Information Technology, Mahatma Gandhi Institute of Technology, Hyderabad, Telangana, India,; 2 Department of Computer Science and Engineering, Chaitanya Bharathi Institute of Technology (A), Gandipet, Hyderabad, India; 3 Department of MCA, Chaitanya Bharathi Institute of Technology, Hyderabad, India; 4 Department of AIML, Chaitanya Bharathi Institute of Technology (A), Gandipet, Hyderabad, India; 5 Department of Information Technology, Chaitanya Bharathi Institute of Technology (A), Gandipet, Hyderabad, India; 6 Department of Information Technology, College of Computer and Information Sciences, Princess Nourah bint Abdulrahman University, Riyadh, Saudi Arabia; 7 Faculty of Computing and IT, Sohar University, Sohar, Sultanate of Oman; 8 Jadara Research Center, Jadara University, Irbid, Jordan; 9 Symbiosis Institute of Technology, Hyderabad Campus, Symbiosis International (Deemed University), Pune, India; King Fahd University of Petroleum & Minerals, SAUDI ARABIA

## Abstract

Terahertz (THz) communication is a promising enabler for next-generation wireless networks because it can support ultra-high data rates. However, severe path loss, molecular absorption, and high sensitivity to blockage significantly limit coverage and reliability. To address these challenges, this work proposes a RIS-assisted UAV positioning (RAVP) framework that integrates reconfigurable intelligent surfaces (RIS) with unmanned aerial vehicles (UAVs) and jointly optimizes RIS configuration and UAV deployment to enhance THz communications. RISs provide controllable reflections to improve propagation conditions, while UAVs enable flexible placement of RISs at advantageous locations. A reinforcement learning (RL)-based strategy that combines modified K-means clustering with gradient-based optimization coordinates user grouping, RIS phase-shift adaptation, and UAV positioning within a unified framework. Simulation results show consistent gains in link robustness, achievable data rate, and user connectivity across different network configurations compared with conventional THz systems without RISs or UAV-assisted optimization. These findings highlight the potential of coordinated RIS-UAV optimization for future 6G-enabled wireless networks, including smart-city and Internet of Things (IoT) applications.

## Introduction

Wireless data demand is increasing rapidly, driving research toward new frequency bands that can provide higher data rates and more reliable connectivity. THz communication, operating in the frequency range of 0.1–10 THz, offers extremely large bandwidth and can enable ultra-high-speed data transmission beyond the capabilities of conventional radio-frequency (RF) systems [1]. These features make THz communication suitable for applications such as real-time 8K video streaming, low-latency cloud services, remote healthcare monitoring, and advanced sensing systems [2].

Despite these advantages, THz communication faces significant technical challenges. A major limitation is severe signal attenuation caused by molecular absorption and free-space path loss. These effects reduce transmission range and link reliability, particularly in outdoor and dense environments where obstacles can block or scatter signals [3]. As a result, maintaining stable links under practical deployment conditions remains difficult, which limits real-world adoption.

### Challenges

Deploying THz systems in real-world environments introduces several challenges. Due to the high operating frequency, THz signals experience high propagation loss and strong reflections compared to lower-frequency waves. This limits communication range and creates coverage gaps. Physical obstacles such as buildings, trees, and vehicles can obstruct line-of-sight (LoS) links, leading to frequent interruptions. User mobility further complicates operation because the topology changes dynamically and direct links become difficult to maintain. In addition, variations in weather and temperature can affect signal propagation characteristics [4].

To address these challenges, adaptive and intelligent solutions are required. RIS and UAV technologies have emerged as promising options. RIS can manipulate propagation through programmable phase adjustments, while UAVs can reposition dynamically to act as aerial relays. Their joint use can improve coverage, enhance signal quality, and increase adaptability to varying network conditions [5].

### Problem statement

Despite the promise of THz communication for ultra-high-data-rate wireless systems, practical deployment remains constrained by unresolved technical limitations. THz signals experience severe distance-dependent path loss, strong molecular absorption, and extreme sensitivity to blockage, which collectively lead to intermittent links and fragmented coverage in realistic environments. Existing mitigation approaches-including static or heuristically placed RISs and preplanned UAV deployments-often cannot adapt to rapid variations in user distribution, channel conditions, and blockage dynamics. Moreover, many optimization strategies treat user grouping, RIS configuration, and UAV trajectory planning as separate (or loosely coupled) problems, which can yield suboptimal overall performance. These limitations prevent conventional THz systems from achieving reliable connectivity and consistent quality of service, particularly in infrastructure-scarce or highly dynamic scenarios. Therefore, a coordinated and adaptive optimization framework is needed to jointly address propagation control, mobility management, and user association in THz networks.

### Motivation

The growing demand for data-intensive and latency-sensitive applications has intensified interest in THz communication as a key enabler for future wireless networks. However, the propagation vulnerabilities of THz signals limit the effectiveness of solutions that rely on fixed infrastructure or static optimization assumptions. While RIS technology provides programmable control over reflections and UAVs provide mobility for flexible network support, their potential is often underutilized due to limited adaptability and a lack of intelligent coordination. In particular, conventional deployment methods may not respond effectively to time-varying channel conditions, user mobility, and blockage-induced link degradation. RL offers a principled way to address these challenges by enabling continual learning and decision-making based on environmental feedback. By jointly optimizing UAV positioning, RIS phase configuration, and user grouping within an RL-driven framework, the network can adapt to changing conditions, improve link robustness, and increase spectral efficiency. This motivates the development of an integrated RAVP framework for reliable, high-performance THz communication in dynamic environments.

## Contributions

In light of the above discussion, this work introduces a novel RAVP framework that addresses key limitations of existing THz communication systems through coordinated and adaptive optimization. The proposed approach jointly handles user association, RIS phase adjustment, and UAV positioning within a unified RL-based framework, enabling real-time adaptation to dynamic network conditions. The main contributions of this paper are summarized as follows:

- An intelligent RIS and UAV-assisted THz communication framework is proposed to mitigate signal blockage, coverage fragmentation, and reliability challenges in high-frequency wireless environments.
- A joint optimization strategy is developed by integrating RL with modified K-means clustering and gradient-based optimization to simultaneously perform user grouping, RIS phase configuration, and UAV positioning.
- Extensive performance evaluation demonstrates enhanced data rates, improved signal quality, and increased system efficiency, highlighting the effectiveness of the proposed RAVP framework for 6G and future wireless networks.

The remainder of this paper is organized as follows. The literature review summarizes related work. The system model section describes the proposed RIS-UAV system model and RL framework. The results and discussion section presents the simulation results and their implications. Finally, the conclusion summarizes the key findings and outlines future research directions.

## Literature review

The literature highlights the challenge of providing wide-area connectivity in satellite-based IoT networks, particularly in terms of efficient spectrum utilization. One proposed solution is a non-orthogonal, slotted-ALOHA multiple-access scheme for satellite communications. This approach supports large-scale device access under limited bandwidth conditions. Although it may introduce additional transmission delay, it provides a practical mechanism for connecting many IoT devices in regions where terrestrial infrastructure is unavailable. Satellite-assisted communication can therefore extend network coverage to remote and underserved areas [5,6].

### IoT applications

UAVs play a significant role in data collection and environmental monitoring because of their relatively low operating cost. Advances in onboard hardware and image-processing techniques have expanded UAV applications, particularly in agriculture. When UAVs and sensors are integrated through IoT frameworks, the collected data can be transmitted over the Internet, enabling better decision-making and improved operational efficiency [7,21].

This study examines the integration of UAVs into 5G new radio networks to enhance wireless communication (WC) capabilities. The focus is on providing reliable and cost-effective connectivity for IoT devices, especially in scenarios where conventional cellular infrastructure is unavailable. Experimental results demonstrate the effectiveness of the UAV-assisted framework in delivering seamless wireless connectivity, highlighting its potential to complement existing 5G wireless networks [8,24].

The emergence of 6G-IoT promises unprecedented advancements in network capacity and management. This work explores the potential of 6G-IoT and ongoing research efforts aimed at realizing its capabilities. Owing to its transformative features, 6G-IoT is expected to play a central role in shaping the future of connected systems [9,15,25].

### Emerging UAV-assisted 6G networks

In 6G networks, UAVs play a critical role in WC; however, operating in open environments and relying on LoS links introduce security vulnerabilities. To enhance security, a three-dimensional beamforming approach with physical-layer support is employed in UAV systems. This method improves the average secrecy rate using an unsupervised neural network based on a Denoising Autoencoder (DAE). By adaptively adjusting beam directions and incorporating artificial noise, the approach enhances communication security in UAV-enabled 6G systems [10,11].

The integration of UAVs with Artificial Intelligence (AI) establishes a robust foundation for edge computing in vehicular environments. This architecture enables a smart and adaptive Vehicle-to-Everything (VoE) network operating in three-dimensional (3D) space. It addresses challenges such as fluctuating network conditions, high computational demands, and large data volumes generated by mobile vehicles. Since satellite networks often experience high latency and limited throughput, UAVs offer extended 3D coverage beyond fixed terrestrial networks, which is particularly relevant for future 6G deployments [10,11].

A real-world experimental evaluation using a 5G radio system demonstrates the performance of UAV-based communication under practical conditions. The network is divided into two slices: one dedicated to UAV control signaling and another for data transmission. The results show that network slicing effectively provides isolated and reliable communication channels for UAV operations [12,13].

Another approach integrates visible light communication (VLC), UAVs, and RISs to improve energy efficiency in wireless networks. In this framework, UAVs serve dual roles by providing network coverage and illumination, while RISs are strategically deployed to reflect signals and enhance connectivity. This combination improves energy utilization and strengthens communication links between UAVs and ground users [14–17].

In UAV-based systems, data management is commonly supported by a distributed database that interfaces with multiple external components through a unified platform. This architecture facilitates efficient data exchange across the network. As UAV operations require reliable and stable communication links, current 5G technologies are considered suitable for supporting both UAV-to-ground and UAV-to-UAV communications [18,19].

The large-scale deployment of Unmanned Autonomous Intelligent Systems (UAIS) in conjunction with 6G Non-Terrestrial Networks (NTN) enables autonomous interactions among self-driving vehicles, UAVs, and robotic systems for applications such as precision agriculture and disaster response. Ongoing research in 6G technologies aims to integrate connectivity and intelligence more seamlessly. Consequently, a comprehensive review of 6G NTN from a UAIS perspective is essential for supporting future developments in this domain [20,21].

### Millimeter-wave spectrum band

This work introduces a passive relay architecture that steers signals in different directions based on frequency characteristics. Using this approach, UAVs can exploit the 5G millimeter-wave (mmWave) spectrum to transmit multiple directional beams, with each beam operating on a distinct channel. This enables simultaneous coverage of multiple regions. The proposed design relies solely on passive components, eliminating the need for complex and costly phased-array systems [12].

Future 6G networks aim to surpass the capabilities of 5G by introducing new functionalities such as ambient sensing and enhanced human–machine interaction. These advancements are driven by the integration of AI and emerging technologies, including THz communication, three-dimensional networking, quantum communication, holographic beamforming, backscatter communication, intelligent reflecting surfaces, and smart caching. Collectively, these innovations are expected to redefine next-generation WC systems [10].

### Modulation techniques

The modulation techniques adopted in this framework comply with established network standards. These schemes dynamically adapt to user requirements, enabling efficient communication while adhering to predefined user configurations and latency constraints. Such flexibility enables effective management of core communication functions through passive processing mechanisms, even in interference-prone environments.

### Data privacy

A layer-wise review methodology enables system designers and network operators to identify vulnerabilities within communication protocols used in 6G applications. By analyzing each protocol layer independently, this approach provides a structured understanding of privacy and security challenges while highlighting areas requiring further investigation. The findings emphasize the importance of strengthening security and data protection mechanisms to address evolving threats in 6G networks [14].

Maintaining secure and efficient communication requires careful examination of the impact of security mechanisms on system latency. Addressing these requirements necessitates robust security models that support controlled system access and efficient resource utilization. In parallel, the expansion of the Internet of Everything (IoE) introduces additional challenges, underscoring the need for intelligent and distributed security solutions suitable for resource-constrained devices [19].

Privacy and security are fundamental to the reliability of next-generation wireless networks and must be incorporated during the early stages of 6G design. For instance, Internet of Vehicles applications introduce new privacy and security concerns affecting road users. Accordingly, comprehensive frameworks are required to protect both system architecture and user data [20].

### Wireless communication

Wireless communication is central to enabling diverse UAV applications and has attracted significant research interest. While conventional technologies such as direct links, Wi-Fi, and satellite communication remain relevant in remote areas lacking cellular coverage, emerging 5G and beyond cellular networks offer scalable and cost-effective solutions for supporting the growing number of UAVs [16].

The realization of telepresence and mixed-reality applications is supported by advances in high-resolution imaging, sensing technologies, accurate positioning systems, and next-generation wireless networks. These developments are expected to drive a transition from traditional smartphones to immersive extended-reality platforms delivered through lightweight wearable devices. Such systems will offer enhanced resolution, frame rates, and dynamic range, enabling more interactive and immersive user experiences [22].

The Internet of Everything facilitates connectivity in remote and underserved regions and represents a key application shaping 6G system architectures. These emerging use cases demand higher data rates and lower latency than current 5G systems, thereby accelerating the evolution toward next-generation WC technologies [23].

### Machine learning in 6G IoT

Machine learning (ML) plays a critical role in enabling intelligent IoT applications across multiple layers, including the application, network, and perception layers. At the application layer, ML techniques are widely used for task offloading and resource allocation, improving system efficiency and scalability. Additionally, edge computing provides localized storage and processing capabilities, facilitating the deployment of edge intelligence. This work presents a comprehensive review of ML applications in IoT systems, emphasizing their role in advancing intelligent IoT environments [24,25]. Furthermore, a detailed survey of ML techniques applied to UAV networks categorizes existing methods based on network and communication characteristics. The study highlights the potential of ML-driven approaches to optimize UAV network performance by leveraging multi-source data and deep learning models [20].

The evolution of 6G networks is expected to introduce AI-driven autonomous systems as a core component. Among various 6G services, video-centric applications are anticipated to dominate data traffic. Key technologies contributing to 6G development include THz communication, AI, optical messaging, three-dimensional networking, UAV integration, and wireless power transfer (WPT). This work examines the combined impact of these technologies on the future landscape of WC [6].

### Recent literature on RIS-UAV-THz communication

Pan *et al.* (2025) [26] introduced an adaptive resource allocation framework for IoT systems operating over RIS-UAV-aided NOMA-enabled THz communication networks. The study integrates computing power networks (CPNs) with intelligent resource management to jointly optimize communication and computation performance. The proposed scheme demonstrates effective improvements in spectral efficiency and task execution reliability under dynamic IoT traffic conditions. However, the work primarily focuses on centralized optimization and assumes ideal channel estimation, which may limit its applicability in highly dynamic UAV-assisted environments with imperfect channel knowledge.

Du *et al.* (2022) [27] investigated the performance limits and optimization strategies of RIS-aided THz communication systems. The authors developed analytical models to characterize path loss, beam misalignment, and reflection efficiency in THz bands, followed by optimization of RIS phase configurations. The results confirm that RIS deployment significantly enhances signal coverage and link reliability in THz networks. Despite its strong theoretical foundation, the study does not consider UAV-assisted mobility or learning-based adaptation, which restricts its applicability to static or semi-static network scenarios.

Pan *et al.* (2025) [28] presented an intelligent resource optimization framework for CPN-enabled IoT systems using RIS-UAV-assisted NOMA-THz communication. The approach employs learning-based optimization to jointly manage communication, computation, and networking resources. Simulation results demonstrate improved system throughput and reduced latency compared to conventional resource allocation schemes. Nevertheless, the framework relies on iterative learning mechanisms with notable computational overhead, which may challenge real-time deployment in large-scale IoT networks.

Song *et al.* (2025) [29] proposed a miniature UAV-aided cooperative THz network incorporating reconfigurable energy harvesting holographic surfaces. The work introduces a novel energy-aware THz communication model that combines cooperative UAV relaying with simultaneous wireless information and WPT. The proposed system achieves enhanced energy efficiency and extended network lifetime. However, the reliance on specialized holographic surfaces and energy harvesting hardware introduces practical deployment constraints and increases system complexity.

**Gaps in existing literature**

- In THz communication systems, signal propagation remains highly vulnerable to blockage caused by obstacles such as buildings and human bodies, leading to significant degradation in link reliability and coverage. Existing solutions do not fully address the need for robust transmission mechanisms under dynamically obstructed environments.
- The increasing deployment of UAVs and IoT devices places substantial pressure on spectrum resources, creating challenges in efficient spectrum allocation and utilization. Current approaches provide limited support for meeting the stringent data rate and latency requirements of emerging UAV-assisted IoT services.
- Although extensive research is underway toward 6G wireless networks, the operational capabilities and performance boundaries of 6G systems remain insufficiently characterized. This uncertainty restricts a clear understanding of how future networks will simultaneously satisfy latency, throughput, and reliability demands.
- While 5G and emerging 6G architectures aim to support heterogeneous services, existing studies offer limited insight into addressing joint latency, throughput, and reliability constraints in UAV-centric and highly dynamic communication scenarios.

S1 Table presents a comparative analysis of representative UAV-enabled IoT and 6G communication frameworks with the proposed approach. Existing works primarily focus on isolated aspects such as data collection efficiency, experimental 5G NR connectivity, secrecy enhancement through beamforming, or energy efficiency using RIS and VLC integration. While these approaches demonstrate notable improvements in specific performance metrics, most rely on fixed configurations, limited adaptability, or partial optimization strategies. In contrast, the proposed framework integrates UAVs, RIS, and THz/mmWave communication within a unified 6G IoT architecture and employs RL for joint optimization of communication and deployment parameters. This enables improved coverage and data-rate performance while addressing adaptability challenges inherent in dynamic 6G environments, albeit with practical hardware constraints.

## Proposed methodology

The proposed model is a unified system that integrates RIS-aided UAV-enabled WPT and THz-enabled multi-IRS-assisted UAV WC. The overall workflow of the proposed approach is illustrated in S1 Fig.

The workflow illustrated in the diagram presents a sequential process designed to enhance the performance of a technical system, particularly in the context of network control. It begins with the *Initialization* phase, during which key system parameters are configured and the required data are loaded. This phase establishes the basis for subsequent operations.

In the *Group Update Iteration* step, user groups are iteratively reviewed and updated to improve system performance. The adjustments performed in this stage support the formation of more efficient group structures, thereby contributing to smoother system operation. Subsequently, the *Optimization Problem Formulation* step defines the system objectives and constraints. This formulation provides a structured framework for achieving key goals, such as enhanced network coverage and reduced energy consumption. After defining the objectives and constraints, the process proceeds to the *User Grouping Optimization* step, where users are organized into optimized clusters to improve resource utilization. The method then focuses on two critical components: *IRS phase-shift optimization* and *UAV location optimization*. By tuning the reflection characteristics of the IRS and optimizing UAV placement, the system enhances signal directionality and coverage, leading to improved network performance.

### RIS-aided UAV-enabled WPT system

The system considers a deployment in which the RIS is mounted on top of a structure. The RIS supports both WC and WPT. The network consists of *N* single-antenna users randomly distributed within the service area. Based on user locations, the area is partitioned into *K* service zones (SZ) to improve system efficiency. Each service zone is served by a UAV, denoted as *U*_*AV*_, which is also equipped with a single antenna. The RIS, UAVs, and users jointly enable WC and WPT across the region. A UAV is an unmanned aerial device capable of autonomous or remote-controlled flight. In WC, UAVs assist in tasks such as monitoring, data collection, and network coverage enhancement. For each service zone, the UAV operates at a fixed altitude *h*_*t*_ to maintain a consistent transmission range *T*_*r*_. The horizontal position of the UAV is defined as $H_{Us}=(x_{hs},y_{hs})$. Compared to variations in altitude *h*_*t*_, horizontal position adjustments introduce fewer stability constraints, as illustrated in S2 Fig.

The RIS is mounted on a wall at a specified height, denoted by *Rh*_*t*_, as illustrated in S3 Fig.

The horizontal placement of the RIS is determined by the coordinates $\left(x_{hs},y_{hs}\right)$. The RIS enhances WC by adaptively adjusting the phase *θ* and amplitude *R* of the incident signal *I*_*S*_. This configuration enables precise control over the reflected signal *S*_*R*_, resulting in improved signal strength *S*_*S*_, reduced interference, and enhanced overall system performance. Here, *I*_*S*_ denotes the signal incident on the RIS.


$$S_{R}=\theta \alpha I_{S}$$

$$S_{S}=\frac{R_{P}}{T_{P}}=|\theta |=\left|e^{j\Phi }\right|$$

The received signal power after reflection by the RIS, denoted *R*_*P*_, is used to evaluate the effectiveness of the RIS in enhancing *W*_*C*_. It represents the signal strength *S*_*S*_ after reflection. In contrast, *T*_*P*_ denotes the power of the transmitted signal incident on the RIS, i.e., the signal strength prior to reflection [5,19].

**Phase Adjustment:**


$$\theta =e^{j\Phi }$$

where *θ* represents the complex reflection coefficient for phase adjustment, and *Φ* denotes the phase shift.

**Amplitude Adjustment:**


$$\alpha =\sqrt{R}$$

where *α* represents the complex reflection coefficient for amplitude adjustment, and *R* denotes the desired amplitude-scaling factor. The term $d_{uavS_{Z},n}$ denotes the distance between the *U*_*AV*_ in service zone *S*_*Z*_ and user n∈N. It is computed as the Euclidean distance between the horizontal position of the *U*_*AV*_, $d_{uavS_{Z}}=(x_{S_{Z},UAV},y_{S_{Z},UAV})$, and the position of user *n*, $P_{n}=(x_{Pn},y_{Pn},0)$, while accounting for the UAV altitude term *Rh*_*t*_ (squared in the distance expression). Accordingly, the distance is expressed as follows [5,19]:


$$d_{uavS_{Z}}=\sqrt{|{wc}_{S_{Z}UAV}-{wc}_{n}{|}^{2}+{{Rh}_{t}}^{2}}$$

This equation computes the straight-line distance between the *U*_*AV*_ and the user by accounting for both horizontal separation and UAV altitude. Based on this formulation, the remaining distances in the system are defined as follows [5,19].


$$d_{UAVS_{Z},R_{IS}}=\sqrt{|w_{S_{Z}UAV}-M_{R}{|}^{2}+(h_{t}-{Rh}_{t}{)}^{2}}$$

Here, $d_{UAVS_{Z},R_{IS}}$ denotes the distance between the RIS and the *U*_*AV*_ operating in *S*_*Z*_.


$$d_{R_{IS}n}=\sqrt{C_{D}|w_{RIS}-{wc}_{n}{|}^{2}+{{Rh}_{t}}^{2}}$$

Similarly, $d_{R_{IS}n}$ represents the distance between the RIS and user *n* in set *N*, where *C*_*D*_ is a constant and the distance remains fixed during the UAV flight.

### THz-enabled multi-IRS-assisted UAV

In addition to the RIS-aided *W*_*PT*_ system, the network supports downlink *D*_*L*_ data transmission using THz frequencies. The system architecture includes a base station (*B*_*S*_) operating at THz frequencies and equipped with multiple antennas. The direct communication channels between the *B*_*S*_ and users in set *N* are obstructed by obstacles such as buildings. Consequently, *D*_*L*_ data transmission is facilitated through airborne IRSs (*A*_*IRS*_). The *A*_*IRS*_ are modeled as uniform planar arrays and assist in reflecting the incident signal *I*_*S*_ to enhance communication performan A uniform planar array is a common antenna configuration in *W*_*C*_ systems. It consists of multiple antenna elements arranged on a two-dimensional grid with uniform spacing. The proposed system integrates RIS-aided *W*_*PT*_ with THz-enabled multi-IRS-assisted *U*_*AV*_-based *W*_*C*_, thereby enabling both WPT and data transmission for users distributed across multiple service areas, as illustrated in S4 Fig.

The total number of reflective elements *E* in the IRS is denoted by $E_{lx}E_{ly}$, where *E*_*lx*_ and *E*_*ly*_ represent the number of elements within the IRS area *A*_*IRS*_ along the X-axis and Y-axis, respectively. The IRS adaptively adjusts the phase shifts *P*_*S*_ of these elements to reflect the incident signal *I*_*S*_, thereby improving the performance of the THz network. For analytical convenience, a relative coordinate system associated with the IRS surface *A*_*IRS*_ is defined by *x*, *y*, *z*, representing the directions along the X, Y, and Z axes, respectively. These coordinates serve as reference points for measuring distances and angles, which enables accurate determination of signal propagation paths and reflection directions. The distances and angles with respect to the reference coordinates *x*, *y*, *z* are computed using standard trigonometric relations. The distance *d* between two points with coordinates $A_{IRS1}=({A_{IRS}x}_{1},{A_{IRS}y}_{1},{A_{IRS}z}_{1})$ and $A_{IRS2}=({A_{IRS}x}_{2},{A_{IRS}y}_{2},{A_{IRS}z}_{2})$ is calculated using the Euclidean distance formula [5,19]:


$$d=\sqrt{(A_{IRS}x_{2}-A_{IRS}x_{1}{)}^{2}+(A_{IRS}y_{2}-A_{IRS}y_{1}{)}^{2}+(A_{IRS}z_{2}-A_{IRS}z_{1}{)}^{2}}.$$

The *U*_*AV*_ is assumed to maintain a constant altitude over the target area. In addition, the energy consumption associated with the movement duration of the *U*_*AV*_ is neglected. These assumptions simplify the analysis of the THz network performance by excluding detailed operational constraints of the *U*_*AV*_. Likewise, the angles between vectors are computed using dot products and inverse trigonometric functions. For instance, the angle *θ* between two vectors *v*_*c*1_ and *v*_*c*2_ is obtained as


$$o=\left\|v_{c1}\right\|\left\|v_{c2}\right\|,$$

$$\theta =\arccos\left(\frac{v_{c1}\cdot v_{c2}}{o}\right).$$

### Time-slot allocation

The system operation relies on a time-division multiple access (TDMA) protocol, as demonstrated in S5 Fig. In particular, the entire operation time indicated as *T*, is split into *S*_*Z*_ + *N* time slots. The interval from 0 to $t_{S_{Z}}$ is allocated for downlink WPT ($D_{L}W_{PT}$), while the remaining time slots are assigned for uplink (*U*_*L*_) wireless data transfer (*W*_*DT*_). In this model, *S*_*WT*_ denotes the *S*_*Z*_-th *W*_*PT*_ time slot, and *N*_*DT*_ represents the *n*^th^ *W*_*DT*_ time slot. Here, *T*_*x*_ and *R*_*x*_ indicate packet transmission and reception, respectively.

The constraints for time-slot allocation are defined as [5,19]:


$$S_{WT}\geq 0,\;\forall s_{z}\in S_{Z}$$

$$N_{DT}\geq 0,\;\forall n\in N$$

$$ak=\sum_{n=1}^{N}N_{DT}$$

$$sw=\sum_{s_{z}=1}^{S_{Z}}S_{WT}$$

T=sw+ak

During the time-slot *S*_*WT*_, the *U*_*AV*_ remains stationary at a predefined location within the $S_{Z}{-}^{th}$ service area and transmits power to all *N* users in that area simultaneously. Since adjusting the transmit power *P*_*t*_ is impractical due to payload and power constraints, it is assumed to be constant. The power received by the *n*-th user in the *S*_*Z*_-th service area is expressed as:


$$\eta h_{tn}=\frac{{\left|{\left(h_{tn}\right)}^{H}⨀2_{n}c_{vS_{Z}}+g_{S_{Z},n}\right|}^{2}}{\sigma^{2}}$$

Here, ${\left|{\left(h_{tn}\right)}^{H}⨀2_{n}c_{vS_{Z}}+g_{S_{Z},n}\right|}^{2}$ represents the composite channel gain between the *U*_*AV*_ and user *n*. The parameter *η* denotes the energy conversion efficiency (0<η≤1), and $⨀2_{n}$ is the diagonal reflection coefficient matrix *R*_*CM*_ of the *D*_*L*_ RIS with a fixed reflection amplitude $\theta_{S_{Z},1,i}$, representing the phase shift of the *i*^*th*^ reflective element. The channels *h*_*tn*_, $c_{vS_{Z}}$, and $g_{S_{Z},n}$ denote the links between the RIS and user *n*, the *U*_*AV*_ and RIS, and the *U*_*AV*_ and user *n*, respectively. Both the *U*_*AV*_ and the user are equipped with single antennas. Due to this single-antenna configuration, the channels associated with the *D*_*L*_ and *U*_*L*_ links are expressed using the same formulation.

During the time-slot *N*_*DT*_, user *n* transmits data to the *U*_*AV*_. As each time slot operates independently, inter-user interference is neglected. The power allocated to user *n* is denoted by *P*_*tn*_ in the *U*_*L*_ *W*_*DT*_ phase. Accordingly, the data rate *d*_*rn*_ from user *n* to the *U*_*AV*_ is given by [5,19]:


$$d_{rn}=\eta h_{n}\log_{2}\left(1+\frac{T_{nI}}{T}\frac{{\left|{\left(h_{tn}\right)}^{H}⨀2_{n}c_{vS_{Z}}+g_{S_{Z},n}\right|}^{2}P_{tn}}{2\sigma^{2}}\right)$$

Here, $⨀2_{n}=\text{diag}(e^{j\theta_{k,1}},\ldots ,e^{j\theta_{k,M}})$ represents the RIS reflection matrix *R*_*CM*_, $2\sigma^{2}$ denotes the noise power, and *P*_*S*_ corresponds to the power consumed by the RIS for information processing. To maintain the self-sustainability of the *W*_*PT*_ structure, the energy consumed by user *n* during the *U*_*L*_ *W*_*DT*_ phase must not exceed the energy harvested during the *D*_*L*_ *W*_*PT*_ phase. Therefore, the energy constraint *e*_*c*_ is defined as:


$$e_{c}=\eta \frac{{\left|{\left(h_{tn}\right)}^{H}⨀2_{n}c_{vS_{Z}}+g_{S_{Z},n}\right|}^{2}P_{tn}S_{WT}}{P_{2n}}\geq T_{nI},\;\forall s_{z}\in S_{Z},\forall n\in N$$

Since, the RIS is installed on building rooftops and the *U*_*AV*_ operates at a high altitude, the transmission between them experiences minimal obstruction. Hence, a LoS channel model is adopted for the channel vector *c*_*v*_ between the *U*_*AV*_ and the RIS, expressed as:


$$c_{v}=\frac{\beta_{0}}{(d_{S_{Z}}^{UR}{)}^{2}}{\left[1,e^{-j2\pi \frac{d\Phi_{S_{Z}}^{UAVR}}{\lambda c}},\ldots ,e^{-j2\pi (M-1)\frac{d\Phi_{S_{Z}}^{UAVR}}{\lambda c}}\right]}^{T}$$

Here, $\beta_{0}$ represents the channel power gain at the reference distance *d*_0_ = 1 m, $\Phi_{S_{Z}}^{UAVR}=\frac{x_{R}-x_{S_{Z}UAV}}{(d_{S_{Z}}^{UAVR}{)}^{2}}$, *d* denotes the antenna spacing, and λc is the carrier wavelength. For the links between the *U*_*AV*_ and users, as well as between the RIS and users, a Rician fading channel model is employed to capture the effects of scattering in the user vicinity. This model incorporates both LoS and non-line-of-sight (NLoS) components, reflecting the propagation conditions in WC environments. Accordingly, the channels *g*_*s*,*n*_ and *h*_*n*_ are modeled as [5,19]:


$$g_{s,n}=\frac{\beta_{0}}{(d_{s_{Z}n}^{UG}{)}^{\alpha_{UAVns_{z}}}}\left(\frac{nR_{S_{Z}}^{UAV}}{nR_{S_{Z}}^{UAV}+1}h_{n}^{LOS}+\frac{1}{nR_{S_{Z}}^{UAV}+1}h_{n}^{NLOS}\right)$$

$$h_{k}=\frac{\beta_{0}}{(d_{s_{Z}n}^{RG}{)}^{\alpha_{Rns_{z}}}}\left(\frac{nR_{S_{Z}}^{R}}{nR_{S_{Z}}^{R}+1}h_{n}^{LOS}+\frac{1}{nR_{S_{Z}}^{R}+1}h_{n}^{NLOS}\right)$$

In the context of WC, CN denotes complex Gaussian noise. The terms $h_{n}^{NLOS}\sim CN(0,1)$ and $h_{n}^{LOS}\sim CN(0,𝐈)$ represent the scattering and deterministic LoS components, respectively, where


$$h_{n}^{LOS}={\left[1,e^{-j2\pi \frac{d\Phi_{URS}}{\lambda c}},\ldots ,e^{-j2\pi (M-1)\frac{d\Phi_{URS}}{\lambda c}}\right]}^{T}.$$

The parameters $\alpha_{UAVns_{z}}$ and $\alpha_{Rns_{z}}$ denote the path loss exponents, while $d_{s_{Z}n}^{UG}$ and $d_{s_{Z}n}^{RG}$ represent the corresponding propagation distances. These parameters characterize the attenuation of signal power with distance in WC systems. Without loss of generality, the base station *B*_*S*_ is located at $q_{b}=(x_{BS},y_{BS},h_{BS})=(0,0,h_{BS})$, while the locations of the users and the *U*_*AV*_ are defined accordingly.

### Channel model

Spreading loss and molecular absorption are the primary factors influencing signal propagation in the THz band. Spreading loss arises from geometric wavefront expansion during propagation, which reduces received power. Molecular absorption results from interactions between THz waves and atmospheric molecules, causing frequency-dependent attenuation. Together, these effects restrict communication range and link reliability in THz-based systems. The channel transfer function is expressed as [5,19]:


$$H(f,d)=\frac{c}{4\pi fd}e^{-0.5k(f)d}$$

In this expression, *f* denotes the carrier frequency, *c* represents the speed of light, and $e^{-0.5k(f)d}$ captures the path-loss effect due to molecular absorption. The carrier frequency of the IRS corresponds to the frequency of the electromagnetic wave manipulated by the IRS for signal reflection and enhancement. This frequency determines the operational range of the IRS and directly affects its ability to improve *W*_*C*_ links. The absorption loss coefficient *k*(*f*) depends on the transmission frequency and is given by:


$$k(f)=\frac{P}{P_{STP}}\frac{T_{STP}}{T}\sum_{Q_{i,g}}\sigma_{i,g}(f)$$

Here, *P*_*STP*_ and *T*_*STP*_ denote the standard pressure and temperature, respectively. *P* and *T* represent the ambient pressure and temperature, *Q*_*i*,*g*_ characterizes the propagation environment, and $\sigma_{i,g}(f)$ denotes the molecular absorption coefficient at frequency *f*.

The total propagation distance *d* is expressed as the sum of two segments, $d=d_{1}+d_{2}$, where *d*_1_ denotes the distance between the base station *B*_*S*_ and the aerial IRS *A*_*IRS*_, and *d*_2_ denotes the distance between the *A*_*IRS*_ and the user *N*. These distances are computed as [5,19]:


$$d_{1}=\sqrt{(x_{BS}-x_{AIRS}{)}^{2}+(y_{BS}-y_{AIRS}{)}^{2}+(h_{BS}-h_{AIRS}{)}^{2}}$$

$$d_{2}=\sqrt{(x_{AIRS}-x_{N}{)}^{2}+(y_{AIRS}-y_{N}{)}^{2}+(h_{AIRS}-h_{N}{)}^{2}}$$

Direct links between the *B*_*S*_ and the users are assumed to be blocked. Consequently, aerial platforms such as the *A*_*IRS*_ are employed to establish reliable wireless links for data transmission. An IRS mounted parallel to a hovering UAV is considered, with its reflecting elements aligned along the x- and y-axes. Let *N*_*x*_ and *N*_*y*_ denote the number of reflecting elements along each axis, resulting in a total of $N_{x}\times N_{y}$ IRS elements. The channel gain *C*_*G*_ of the cascaded link involving the *B*_*S*_, *A*_*IRS*_, and user *N* is denoted by *g*(*t*). The IRS beamforming matrix *θ* is defined as [5,19]:


$$\theta =diag\left(e^{j\Phi_{1}},\ldots ,e^{j\Phi_{M}}\right)$$

Each diagonal entry corresponds to the reflection coefficient $\omega_{n}$ of the *n*-th IRS element, where $\omega_{n}\in (0,1)$ and *j* denotes the imaginary unit. For analytical simplicity, $\omega_{n}=1$ is assumed, and the phase shift Φ∈(0,2π). The cascaded channel gain from the *B*_*S*_ to the *A*_*IRS*_ and then to the user *N* at time *t* is expressed as:


$$g(t)=\frac{c}{8\sqrt{\pi^{3}f_{r}u(t)r_{o}(t)}}e^{-j2\pi f(r_{t}(t)+r_{o}(t))}e^{-0.5k(f)(r_{t}(t)+r_{o}(t))}$$

Here, *r*_*t*_(*t*) represents the propagation distance from the *B*_*S*_ to the *A*_*IRS*_, and *r*_*o*_(*t*) denotes the distance from the *A*_*IRS*_ to the user. The relative phase difference between the signal received from the *B*_*S*_ and the reference IRS element is given by:


$$\theta_{N_{x},N_{y}}(t)=\frac{2\pi fr_{t}(t{)}_{1}}{r_{t}(t)r_{N_{x},N_{y}}(t)}$$

where $r_{N_{x},N_{y}}(t)$ denotes the relative position vector of the IRS element. The array response vector from the *B*_*S*_ to the IRS at time *t* is defined as:


$$A_{V}=\left[e^{-j\theta_{1}},\ldots ,e^{-j\theta_{M}}\right]$$

The reflected signal phases are jointly adjusted to enable directional beamforming toward the intended users. Accordingly, the transmit array vector from the *A*_*IRS*_ to the *k*^th^ user is expressed as [5,19]:


$$e_{i}=\left[e^{-jB_{1}},\ldots ,e^{-jB_{M}}\right]$$

All multi-IRS-enabled UAVs operate within the same frequency band, which introduces co-channel interference denoted by *ψ*. The signal-to-interference-plus-noise ratio (*S*_*INR*_) at a user served by a UAV at time *t* is given by:


$$S_{INR}=\frac{1+P_{t}G_{t}}{\psi +B_{i}\frac{de^{0.5k(f)d}}{\sigma^{2}}}$$

Here, *B*_*i*_ denotes the allocated bandwidth, and $\sigma^{2}$ represents the additive white Gaussian noise power. The achievable data rate *R*_*m*_ of user *R*_*m*_, derived from the corresponding *S*_*INR*_, is expressed as:


$$R_{m}=\frac{B_{i}}{m}\log_{2}\left(\frac{1+P_{t}G_{t}}{\psi +B_{w}\frac{de^{0.5k(f)d}}{\sigma^{2}}}\right)$$

To address the complexity of the considered communication system, the original optimization problem is decomposed into tractable subproblems. The proposed framework adopts a two-stage strategy: first, optimal user grouping is performed; second, joint optimization of the transmit power *P*_*S*_ and UAV placement is carried out to maximize the average system data rate. This decomposition improves computational efficiency while enhancing overall network performance in terms of *d*_*rn*_.

### K-means grouping

This section describes a step-by-step procedure for improving user grouping in the network. The objective is to iteratively refine the group configuration until convergence. The method focuses on enhancing the value of *d*_*rn*_ while satisfying the associated constraints. To simplify the optimization process, the overall problem is decomposed into smaller subproblems. Initially, users are grouped using an improved variant of the K-means clustering algorithm.

The core principle of this clustering strategy is to partition users into distinct groups based on spatial proximity and the *d*_*rn*_ constraints. In large-scale THz communication scenarios, managing a high number of users becomes challenging. To address this issue, K-means clustering is applied to group users according to minimum distance and rate requirements. This strategy reduces computational complexity and improves the effectiveness of user grouping. K-means is well suited to large-scale, unlabeled datasets and is widely used due to its simplicity, fast convergence, and low computational overhead (Ref. Algorithm 2 and Algorithm 3). In the proposed implementation, the initial user clusters are selected carefully to accelerate convergence and improve clustering stability.

**Algorithm 1** User Grouping Algorithm

**Require:** Number of users, distance threshold, *d*_*rn*_ threshold.

**Ensure:** Optimal user groups.

 1: **Initialization:** Randomly generate user locations and initialize *d*_*rn*_ for each user.

 2: **Group update iteration:**

 3: **For** each user *m* = 1 to *M* **do**

 4:   Estimate distance to neighboring users.

 5:   Assign random *d*_*rn*_ for each user.

 6:   Assign the user to the group with the smallest *d* and the largest *d*_*rn*_ within the cluster.

 7: **End For**

 8: Repeat steps for all users until convergence.

At each iteration, the Algorithm 1 identifies the minimum distance *d* between user *n* and its neighbors. A user with the smallest distance to a neighbor is assigned to the group served by *A*_*IRS*_. Each user is initially assigned a random distance value *d*_*rn*_. Group formation is then refined based on the aggregate *d*_*rn*_ values within each group to improve cluster compactness. Subsequently, the cluster coverage probability is evaluated to verify the suitability of the resulting groups. The target area *T*_*r*_ is computed using the altitude of *A*_*IRS*_ and the beam angle *θ* of the *U*_*AV*_ in the THz communication setup, as given by [5,19]:


$$T_{r}=h_{tj}\tan(\theta )$$

In this expression, *θ* denotes the half-power beam angle, corresponding to the narrow radiation width of the antenna.

**Algorithm 2 K-means Clustering**

 1: **Initialization:** Select centroids randomly from the dataset: $C=\{c_{1},\ldots ,c_{n}\}$.

 2: **Assignment:** Assign each point *x*_*i*_ to the nearest centroid *c*_*j*_ based on distance: $\text{argmin}||x_{i}-c_{j}|{|}^{2}$.

 3: **Update Centroids:** Update centroid positions by computing the mean of data points assigned to each centroid:

          $c_{j}=\frac{1}{\left||s_{j}|\right|}\sum_{x_{i}\in s_{j}}x_{i}$

 where *s*_*j*_ represents the set of data points allocated to centroid *c*_*j*_.

 4: **Repeat:** Repeat until a stopping criterion is met.

 5: **Optimal Grouping:** Once convergence is achieved, the data points are grouped based on the final centroid positions.

*Therefore, the problem of maximizing d*_*rn*_ *can be expressed as follows:*


$$\max_{\left(\theta_{n},q,p,\omega_{n}\right)}\sum_{m=1}^{M}R_{m}$$

*subject to*


$$R_{m}\geq R_{\min},\forall t\in k$$

$$\sum_{m=1}^{M}P_{k}\leq P_{\max}$$

$$\omega_{n}=1,\forall n\in N$$

$$0\leq \theta_{n}\leq 2\pi ,\;n=1,\ldots ,N$$

$$q(x,y)\in f_{a}$$

**Algorithm 3** Deterministic Rules Estimation

 1: Execute the selected action *a*_*t*_ and observe the next state *s*_*t*+1_ and reward *r*_*t*_.

 2: Store the transition $\left(s_{t},a_{t},r_{t},r_{t+1}\right)$ in the experience replay buffer *R*.

 3: Sample a mini-batch of transitions $\left(s_{t},a_{t},r_{t},r_{t+1}\right)$ from the replay buffer *R*.

 4: Estimate the target Q-value *y*_*i*_ using the target critic network:

       $y_{i}=r_{i}+\gamma Q^{\prime }(s_{t+1},\mu^{\prime }(\mu (s_{t+1}|\theta {)}^{\prime }))$

 5: Update the critic network $Q(s,a|\theta^{q})$ by minimizing the loss function:

       $L=y_{i}+\frac{1}{N}\sum_{i}\Delta_{a}^{2}Q(s,a|\theta^{\mu })$

 6: Update the actor network $\mu (s|\theta^{\mu })$ using the tested *R*_*G*_:

       $\Delta \theta^{\mu }=\frac{1}{N}\sum_{i}Q_{i}(s,a|\theta^{Q}{)}^{2}s=s,a=\Delta \theta^{\mu }(s|\theta^{\mu })s_{i}$

 7: Update the actor parameters $\theta^{\mu }$ using the sampled *R*_*G*_.

 8: Update the target networks $\mu (s|\theta^{\mu })$ and $\sum_{i}Q_{i}(s,a|\theta^{Q})$ using soft updates:

       $Q_{\mu }^{\prime }=\tau \theta^{\mu }+(1-\theta^{\prime \mu })$

       $\theta^{\prime Q}=\alpha^{Q}+(1-\theta^{\prime Q})$

 9: Repeat steps until convergence or a predefined number of iterations.

Here, the constraint ($R_{m}\geq R_{\min},\forall t\in k$) guarantees the minimum achievable rate for all *N* users, thereby satisfying the quality requirements for each *n*. The constraint $\sum_{m=1}^{M}P_{k}\leq P_{\max}$ limits the total transmit power *T*_*P*_ so that it does not exceed the maximum allowable power budget. The condition ($\omega_{n}=1,\;\forall n\in N$) indicates that each IRS reflecting element operates with full reflection, where the reflection coefficient magnitude of all IRS elements is set to unity. The constraints ($0\leq \theta_{n}\leq 2\pi ,\;n=1,\ldots ,N$) describe the phase-shift characteristics of the IRS reflecting matrices. Each matrix corresponds to an IRS reflecting matrix *P*_*S*_, implying that all incident signals are reflected without power loss, thereby improving signal transmission efficiency. The restriction ($q(x,y)\in f_{a}$) confines the UAV trajectory to a predefined feasible region. The optimization problem in ($maximize(\theta_{n},q,p,\omega_{n})\sum_{m=1}^{M}R_{m}$) is non-convex and challenging to solve directly. Hence, the original problem is decomposed into sequential subproblems, focusing on optimal user clustering and the joint optimization of UAV locations and IRS configurations.

This framework provides a systematic way to select the *U*_*AV*_ location and IRS placement *P*_*S*_ to enhance system performance. By employing RL techniques, the model adapts to varying network conditions, improves performance, and reduces power usage and computational cost. The framework consists of a state *s*_*t*_, an action *a*_*t*_, a reward *r*_*t*_, and an IRS-*U*_*AV*_ agent. The agent interacts with the environment by taking actions, receiving rewards, and updating its state accordingly. Rewards provide feedback that guides the agent toward actions that yield better outcomes. Through repeated interactions, the agent progressively improves its decision-making capability.

In RL, actions are selected sequentially to maximize cumulative reward under varying conditions. The agent observes the current state and chooses an action that is expected to yield a higher reward. Through repeated actions and feedback, the agent accumulates experience and gradually refines its policy, improving performance over time. The integrated design combines UAVs and RISs to improve energy efficiency in wireless links. In RL, the state represents the current environment observed by the agent and contains the information required for decision-making. The state at time *t*, denoted by *s*_*t*_, consists of the IRS placement *P*_*S*_ and the *U*_*AV*_ position. Let *t*_*i*_ = *t* represent the previous UAV horizontal position.


$$a=\left[x_{\left(t_{i}-1\right)},\;y_{\left(t_{i}-1\right)}\right]$$

$$s_{t}=[\theta_{\left(ti+1\right)},\theta_{(ti-1)2N},a]$$

The action defines the set of operations available to the agent at a given time step and represents possible changes to the current system configuration. The action taken at time *t* denoted by *a*_*t*_, includes the IRS configuration *P*_*S*_ and the movement of the *U*_*AV*_ [5,19]:


$$a_{t}=[\theta_{(t)1},\ldots ,\theta_{(t)2N},x_{\left(t\right)},y_{\left(t\right)}]$$

The reward quantifies the immediate benefit obtained after taking an action in a given state and serves as the basis for learning. After executing action *a*_*t*_ in state *s*_*t*_ at time *t*, the agent receives a reward $r_{t}(s_{t},a_{t})$. The reward corresponds to the aggregated value *d*_*rn*_ across all clusters:


$$r_{t}=r_{t,sum}=\sum_{m=1}^{M}r_{tm}$$

The agent selects actions in a continuous manner, guided by the observed state, to achieve higher rewards. The proposed algorithm enables the agent to learn optimal strategies under dynamically changing THz network conditions.

### Advantages of the proposed method

The proposed optimization framework offers several advantages for THz communication systems in future wireless networks.

- The joint optimization of user grouping, RIS phase configuration, and UAV positioning improves convergence behavior compared to conventional separate or iterative optimization approaches.
- The integration of RL with modified K-means clustering enables adaptive and scalable user association under dynamic network conditions.
- UAV-assisted deployment enhances network coverage and link reliability in blockage-prone THz environments, particularly in infrastructure-limited scenarios.
- RIS-based passive beam control improves signal quality without introducing significant power or hardware overhead.
- The proposed framework is compatible with emerging 6G architectures and supports intelligent, autonomous network operation.

### Limitations of the proposed method

Despite these advantages, the proposed framework has certain limitations that warrant further investigation.

- The RL process introduces additional computational complexity during the training phase, which may impact real-time deployment in highly resource-constrained systems.
- The performance of the framework depends on accurate channel state information and environmental awareness, which can be challenging to maintain in rapidly changing UAV-assisted networks.
- The current study considers a limited number of UAVs and RIS elements, and scalability to ultra-dense deployments requires further validation.
- The optimization framework is evaluated under controlled simulation settings, and real-world implementation may introduce additional constraints such as hardware imperfections and regulatory restrictions.
- Energy consumption associated with UAV mobility is not explicitly optimized and may affect long-duration operations.

## Results and discussion

This section evaluates the effectiveness of the proposed approach using RIS and IRS in communication systems. The analysis is based on simulation results and demonstrates the role of these techniques in enhancing the performance of *W*_*C*_ systems. The integration of RIS and IRS into the network results in noticeable improvements in key performance aspects such as data rate, coverage, and link reliability.

### Simulation setup

This subsection describes the key factors contributing to the effectiveness of RIS and IRS in the proposed framework. By enabling controllable signal reflection, these technologies improve signal propagation, mitigate interference, and support more efficient utilization of network resources. The coordinated integration of RIS and IRS facilitates enhanced spectrum usage and improved communication reliability under dynamic channel conditions.

The performance evaluation was conducted using the proposed RAVP routing and optimization framework. The simulation environment consisted of 101 IoT nodes deployed over a two-dimensional area of 1000×500 m^2^. UAV-assisted RIS elements were adaptively positioned at controlled altitudes to support THz-band communication. The total simulation duration was set to 200 time units. All experiments were executed over multiple Monte Carlo trials with fixed random seeds, and the reported results represent statistically averaged performance metrics, as summarized in S2 Table. S2a Table summarizes the reproducibility and statistical-robustness reporting used in this study.

### Reachable Hops with and without RIS

S6 Fig and S3 Table report the number of reachable hops with and without RIS. The results indicate that using RIS increases the number of reachable hops, implying that the network can maintain connectivity across more nodes and over longer paths. The proposed RIS-assisted approach helps address coverage limitations, extends communication range, and improves communication stability. Overall, these observations show that RIS can enhance the performance of *W*_*C*_ systems, particularly in large-scale and dynamic network environments.

Integrating RIS into the network yields a clear improvement in the number of reachable hops. For example, in the single-user scenario, reachable hops increase from 24 (No RIS) to 87 (RIS). As the number of users increases, reachable hops remain consistently higher with RIS: 77–178 (2 users), 165–292 (3 users), 272–376 (4 users), 362–482 (5 users), 436–563 (6 users), 534–574 (7 users), and 558–594 (8 users). All measurements were obtained over a simulation duration of 200 units. Overall, these results confirm that RIS extends communication paths across varying user configurations.

### Average data rate comparison

S4–S7 Tables and S7–S10 Figs compare the average data rate as a function of the number of users and the number of IRS elements for different protocols. The proposed RAVP consistently achieves the highest data rates among the evaluated schemes. This improvement is primarily attributed to effective RIS utilization and its coordinated operation with other system components. Overall, RIS integration improves signal propagation, reduces interference, and enhances channel conditions, which collectively increase achievable data rates.

The average data rate (bps/Hz) achieved by the proposed RAVP framework consistently exceeds that of existing methods, including PPO, Phase Shift, and Random Phase, across a wide range of user counts. For example, with 10 users, RAVP attains 405 bps/Hz, compared with 387 bps/Hz (PPO), 365 bps/Hz (Phase Shift), and 340 bps/Hz (Random Phase). This advantage persists as the number of users increases, with RAVP achieving 467 bps/Hz (20 users), 503 bps/Hz (30 users), and a peak value of 555 bps/Hz (60 users). Even at 90 users, RAVP maintains a higher average data rate of 530 bps/Hz, outperforming the other approaches across all evaluated scenarios.

S4a Table provides a compact summary of the main performance outcomes (reachable hops, data rate, satisfaction, and signal-quality proxies) extracted from S3–S8 Tables, while explicitly tying the reporting to the simulation protocol in S2 Table. For each highlighted scenario, it identifies a representative baseline and the corresponding RAVP value and states that results are to be reported with mean ± standard deviation and 95% confidence intervals over *N* = 100 independent Monte Carlo trials (with fixed seeds), with additional run-to-run variability reporting for RL-based methods.

### Data rate comparison of existing vs. proposed methods

By exploiting the reflective properties of RIS and strategically integrating it with other network components, the proposed RAVP demonstrates significant improvements in data rate performance compared with existing methods. These results highlight the importance of RIS in enabling high-speed and reliable communication in future wireless networks.

The data rate performance of the proposed RAVP algorithm was evaluated and compared with several existing methods, including the proximal policy optimization (PPO) algorithm with 16-IRS and 64-IRS configurations, the Phase Shift algorithm, and the Random Phase Shift algorithm. For the single-user scenario, the UAV algorithm achieved a data rate of 235 bps/Hz, whereas the proposed RAVP with 16 IRS achieved 402 bps/Hz. When the proposed RAVP algorithm was configured with 64 IRS, it achieved the highest data rate of 451 bps/Hz. As the number of users increased, the proposed RAVP algorithm with 64 IRS consistently outperformed all other algorithms, reaching a maximum data rate of 551 bps/Hz with five users. These results demonstrate the improved efficiency of the proposed RAVP algorithm, particularly when combined with 64 IRS, in enhancing data rate performance across different user scenarios.

S6 Table presents a comparison of data rates (in bps/Hz) under different conditions, including the absence of IRS and the use of various algorithms such as PPO, Phase Shift, Random Phase Shift, and the proposed RAVP. Across all considered scenarios (4, 16, 32, 56, and 64 IRS elements), IRS-assisted configurations consistently achieve higher data rates than the case without IRS. Among the evaluated methods, the proposed RAVP attains the highest data rate in every scenario, followed by the PPO algorithm, the Phase Shift algorithm, and the Random Phase Shift method. Furthermore, data rates generally increase as the number of IRS elements increases, with the proposed RAVP demonstrating the most pronounced performance improvement.

S7 Table compares satisfaction rates for five algorithms-the UAV algorithm, PPO, Phase Shift, Random Phase Shift, and the proposed RAVP-across user counts ranging from 10 to 70. The proposed RAVP consistently achieves the highest satisfaction rate, starting at 0.90 for 10 and 20 users and gradually decreasing to 0.51 for 70 users. In contrast, the other algorithms yield lower satisfaction rates across all scenarios. The UAV algorithm performs worst, particularly as the number of users increases. PPO and Phase Shift show moderate performance, while Random Phase Shift generally outperforms them but remains inferior to RAVP.

S11 Fig and S8 Table compare existing and proposed algorithms using three key metrics: signal propagation, interference mitigation, and channel conditions. Without IRS technology, baseline performance is limited (150 m signal propagation, 10 dB interference mitigation, and 4.0 bps/Hz channel conditions). With IRSs, performance improves substantially. PPO increases signal propagation to 200 m, improves interference mitigation to 15 dB, and enhances channel conditions to 5.5 bps/Hz. Phase Shift further improves these metrics to 250 m, 18 dB, and 6.0 bps/Hz, respectively. Random Phase Shift yields moderate gains (220 m, 16 dB, and 5.7 bps/Hz). The proposed RAVP achieves the best performance across all metrics: 280 m, 20 dB, and 6.5 bps/Hz. Overall, these results demonstrate the effectiveness of IRS-based optimization, with RAVP delivering superior signal quality and improved network efficiency.

S9 Table compares the proposed RAVP framework with representative state-of-the-art methods in RIS- and UAV-assisted THz (or related) wireless networks using aligned metrics from the Results and discussion section. Du *et al.* (2022) [27] provides theoretical insights into RIS-aided THz channels but does not report key metrics such as reachable hops or adaptability under dynamic conditions. Pan *et al.* (2025) [26] and Pan *et al.* (2025) [28] report moderate-to-high data rates using resource allocation and iterative learning, but they do not quantify coverage extension or robustness under dynamic UAV-assisted scenarios. Song *et al.* (2025) [29] emphasizes energy-aware design and improved signal propagation; however, reliance on specialized hardware can constrain scalability. In contrast, the proposed RAVP framework achieves higher reachable hops and average data rates and improves signal propagation, interference mitigation, and channel conditions, while supporting dynamic adaptability and scalability through RL-based joint optimization. Overall, these results demonstrate the performance advantages of RAVP under the simulation conditions considered in this work (S2 Table).

S9a Table reports a quantitative metric comparison between the proposed RAVP coordination strategy and representative recent RIS/THz optimization studies [26–29] under consistent evaluation assumptions (same channel model and system parameterization as documented in S2 Table and averaged over *N* = 100 Monte Carlo trials with fixed seeds tabulated in S2a Table). The reported measures are: (i) *throughput* (spectral efficiency, bps/Hz), (ii) *E2E latency* (ms) capturing scheduling/coordination delay effects, (iii) *reliability* (success probability, equivalently 1−outage ), and (iv) *energy efficiency* (bits/J). In addition, the table explicitly reports the relative gains of RAVP versus the best SOTA entry among [26–29], showing a + 5.7% throughput improvement (555 vs. 525 bps/Hz), an 18.9% latency reduction (15.0 vs. 18.5 ms), and a + 2.1% absolute reliability increase (0.96 vs. 0.94). These significant gains are achieved because RAVP performs *joint* coordination across (a) user grouping (reducing contention and improving link matching), (b) RIS phase adaptation (constructive combining to increase effective channel gain/SINR), and (c) UAV positioning (shorter propagation distances and improved LoS probability). By optimizing these coupled degrees of freedom together, the proposed framework reduces packet retransmissions/outage events (higher reliability), shortens service times (lower latency), and increases the delivered information per unit power (higher energy efficiency), thereby outperforming approaches that optimize only a subset of these components.

S9b Table reports variability measures for the S9a Table results. Specifically, it states the Monte Carlo protocol (*N* = 100 independent trials per point) and summarizes the averaging procedure (mean over trials) with standard deviations and 95% confidence intervals for each key metric. Fixed and documented random-seed initialization is assumed across all methods to enable independent replication. For the RL-based evaluation of the proposed RAVP, multiple independent training runs (five runs with different seeds) are also summarized to quantify run-to-run variability. This separation of Monte Carlo channel randomness from learning stochasticity makes the robustness of the reported gains explicit.

## Discussion

The results provide clear evidence that the proposed RAVP framework enhances the reliability and performance of THz communication systems through coordinated RIS and UAV optimization. Compared with conventional THz setups without RIS or UAV-assisted control, the proposed approach yields consistent improvements across multiple performance dimensions, including coverage, achievable data rate, signal quality, and user satisfaction.

A key observation from the reachable-hops analysis is the substantial expansion of communication paths enabled by RIS integration. The increase in reachable hops across varying user densities indicates that the proposed framework mitigates THz signal blockage and molecular absorption effects. This improvement translates into enhanced network coverage and more robust connectivity, particularly in dense or obstructed environments where conventional THz links suffer from frequent disconnections. Overall, RIS-assisted reflection combined with adaptive UAV positioning provides a practical mechanism for sustaining multi-hop THz communication under dynamic conditions.

The average-data-rate results further highlight the benefits of the proposed joint optimization strategy. Across all evaluated scenarios, the RAVP framework outperforms benchmark approaches such as PPO-based control, phase-shift optimization, and random phase configurations. These gains are attributed to coordinated adjustment of RIS phase shifts and UAV trajectories, which improves channel alignment and reduces attenuation. Unlike static or separately optimized schemes, the joint learning-based design can adapt to changes in user distribution and channel conditions, yielding sustained throughput improvements as network load increases.

The effect of RIS element density on data-rate performance reveals an important scalability characteristic of the proposed approach. As the number of IRS elements increases, the RAVP framework exhibits more pronounced gains than existing methods. This behavior indicates that the proposed optimization strategy effectively exploits the additional spatial degrees of freedom provided by larger RIS configurations. In contrast, baseline methods show diminishing returns under similar conditions, suggesting limited adaptability to increasing system complexity.

Further insights are obtained from the signal-propagation, interference-mitigation, and channel-condition evaluations. The proposed RAVP framework achieves the highest signal-propagation distance and strongest interference suppression among the compared methods. These improvements reflect the ability of the RL-based controller to steer reflected beams and reposition UAVs in response to environmental variation. Improved channel conditions (higher achievable capacity) confirm that the proposed approach enhances link quality rather than merely increasing transmit power or resource usage.

User-satisfaction results provide an application-level perspective on system performance. The consistently higher satisfaction rates achieved by RAVP across all user densities indicate improved fairness and quality-of-service provisioning. As the number of users increases, competing schemes show a sharp decline in satisfaction due to congestion and suboptimal resource allocation. In contrast, the proposed framework maintains relatively stable satisfaction levels, demonstrating its effectiveness in balancing coverage, throughput, and interference under high-load conditions.

Compared with recent state-of-the-art studies [26,29] on RIS-aided THz optimization and UAV-assisted communication, the proposed method offers distinct advantages. Prior works primarily focus on static RIS optimization, centralized resource allocation, or iterative learning mechanisms and often neglect mobility, scalability, or real-time adaptability. The RAVP framework addresses these limitations through a unified RL-based joint optimization strategy that simultaneously considers user grouping, RIS configuration, and UAV positioning. This integrated design improves convergence behavior and enhances system responsiveness in dynamic environments.

Despite these strengths, certain limitations remain. The proposed framework assumes accurate environmental sensing and control signaling between UAVs and RIS units, which may introduce overhead in large-scale deployments. In addition, hardware constraints related to RIS size and UAV endurance may affect practical implementation. These aspects motivate future work on distributed learning strategies, lightweight control signaling, and energy-aware optimization.

## Conclusion and future scope

This study presented a joint RIS-UAV-assisted optimization framework to enhance THz communication performance. The proposed approach enables controllable signal reflections via RIS, improving propagation conditions and reducing interference inherent to high-frequency THz links. UAVs serve as flexible aerial platforms for adaptive RIS deployment, thereby extending network coverage and improving link reliability. An RL-based optimizer that integrates modified K-means clustering and rule-based gradient adjustment jointly addresses user grouping, RIS phase configuration, and UAV positioning. Simulation results demonstrate consistent improvements in coverage, achievable data rate, and signal quality across diverse THz communication scenarios, validating the effectiveness of the proposed framework. Despite these gains, certain limitations remain. The framework relies on RIS hardware with fine-grained phase control and UAV platforms capable of stable, precise positioning, which may introduce deployment complexity and cost constraints in large-scale networks. In addition, the computational overhead associated with learning-based joint optimization may affect scalability under stringent latency requirements. The impact of channel-estimation errors, fast-varying mobility, and dense blockage conditions was also not fully captured, which may influence performance in highly dynamic real-world environments. Future research directions include integrating deep RL to enhance adaptability under rapidly varying channel conditions and user mobility. Practical investigations involving real-time RIS and UAV deployments-such as hardware-in-the-loop experiments and outdoor field trials-can provide deeper insights into implementation feasibility. Further work can also focus on low-cost, energy-efficient RIS architectures and autonomous UAV platforms to improve scalability and support smart-city and IoT-oriented THz communication applications. These directions provide a pathway toward robust, high-performance THz communication systems for next-generation wireless networks.

## Supporting information

S1 Fig Workflow Schematic of the Proposed Approach. (ZIP)

S2 Fig System Architecture of RIS-assisted UAV Communication Network. (ZIP)

S3 Fig Height Optimization of UAV-IRS for Maximized Signal Strength. (ZIP)

S4 Fig IRS placement and coverage area for optimal user communication. (ZIP)

S5 Fig TDMA time-slot allocation in the proposed model. (ZIP)

S6 Fig Reachable Hops Comparison With and Without RIS. (ZIP)

S7 Fig Impact of Number of Users on Average Data Rate (bps/Hz). (ZIP)

S8 Fig Impact of Increasing the Number of Users on Data Rate (bps/Hz). (ZIP)

S9 Fig Impact of IRS on data rate (bps/Hz). (ZIP)

S10 Fig Impact of Number of Users on Satisfaction Rate (%). (ZIP)

S11 Fig Impact of signal propagation, interference mitigation, and channel conditions (existing vs. proposed). (ZIP)

S1 Table Comparative analysis of UAV-enabled IoT and 6G communication frameworks. (ZIP)

S2 Table Comprehensive simulation parameters. (ZIP)

S2a Table Reproducibility and statistical robustness reporting. (ZIP)

S3 Table Reachable hops with and without RIS. (ZIP)

S4 Table Average Data Rate of Existing vs. Proposed Methods. (ZIP)

S4a Table Key results with statistical reporting (based on simulation parameters in S2 Table, *N* = 100 Monte Carlo trials). (ZIP)

S5 Table Data Rate of Existing vs. Proposed Methods (bps/Hz). (ZIP)

S6 Table Data Rate With and Without IRS (bps/Hz). (ZIP)

S7 Table Satisfaction Rate of Existing vs. Proposed Methods. (ZIP)

S8 Table Comparison of signal propagation, interference mitigation, and optimized channel conditions. (ZIP)

S9 Table Comparative analysis of state-of-the-art methods against the proposed RAVP framework using aligned performance metrics. (ZIP)

S9a Table Quantitative metric comparison with recent state-of-the-art methods [26–29]. (ZIP)

S9b Table Statistical reporting and robustness summary for S9a results (*N* = 100 Monte Carlo trials; fixed seeds; RL multi-run variability). (ZIP)

## Code Availability

The simulation code and implementation scripts used in this study are maintained in a private Git repository. Access can be granted for academic and research verification upon reasonable request. A temporary private link is provided below for editorial and review purposes only: https://github.com/PREMKUMARCH/Project-code-RAVP.git (private)

Access to the repository can be enabled upon request to Premkumar Chithaluru.
