# Supplementary figures and images for: Reconfigurable intelligent surface and UAV coordination for reliable THz wireless networks
Source: PLoS One. 2026 Mar 23;21(3):e0345290. doi: 10.1371/journal.pone.0345290 (PMC13008106; doi:10.1371/journal.pone.0345290)

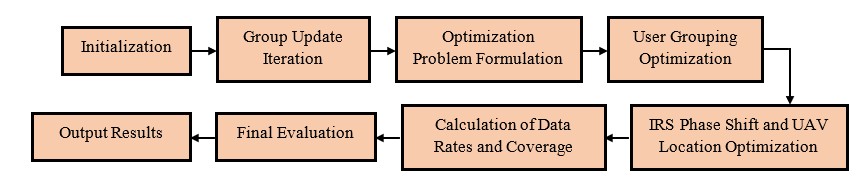

Supplement: S1 Fig — (ZIP) [file pone.0345290.s001.zip › S1_Fig.jpg]

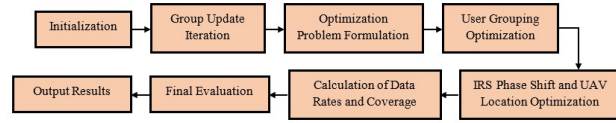

Figure 1: \*  
S1 Fig Workflow Schematic of the Proposed Approach

Supplement: S1 Fig — (ZIP) [file pone.0345290.s001.zip › S1_Fig.pdf]

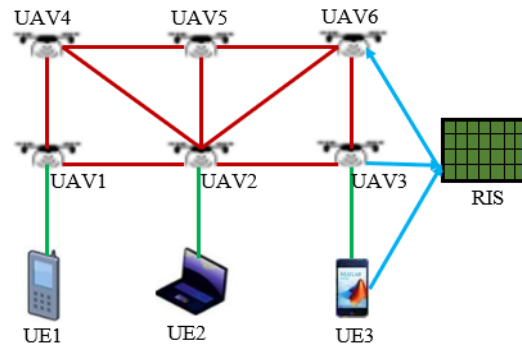

Figure 1: \*  
S2 Fig System Architecture of RIS-assisted UAV Communication Network

Supplement: S2 Fig — (ZIP) [file pone.0345290.s002.zip › S2_Fig.pdf]

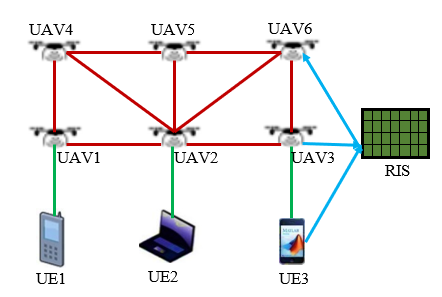

Supplement: S2 Fig — (ZIP) [file pone.0345290.s002.zip › S2_Fig.png]

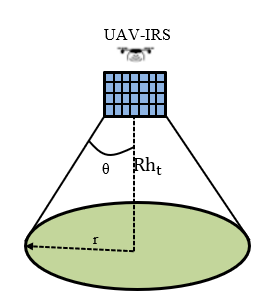

Supplement: S3 Fig — (ZIP) [file pone.0345290.s003.zip › S3_Fig.png]

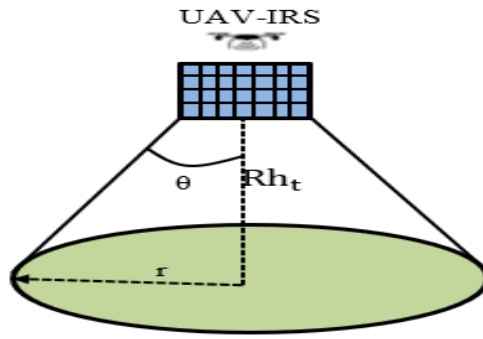

Figure 1: \*  
S3 Fig Height Optimization of UAV-IRS for Maximized Signal Strength

Supplement: S3 Fig — (ZIP) [file pone.0345290.s003.zip › S3_Fig.pdf]

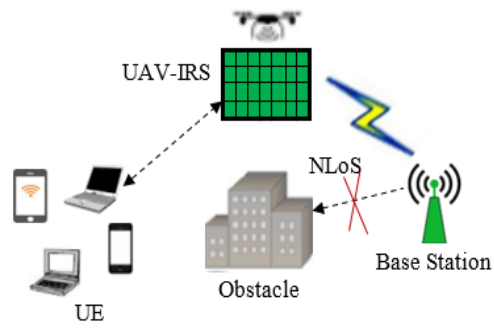

Figure 1: \*  
S4 Fig IRS placement and coverage area for optimal user communication

Supplement: S4 Fig — (ZIP) [file pone.0345290.s004.zip › S4_Fig.pdf]

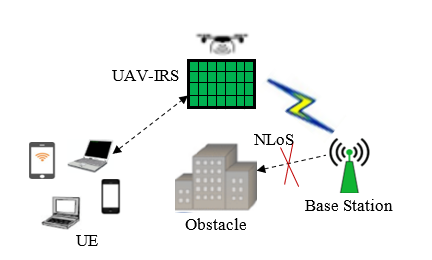

Supplement: S4 Fig — (ZIP) [file pone.0345290.s004.zip › S4_Fig.png]

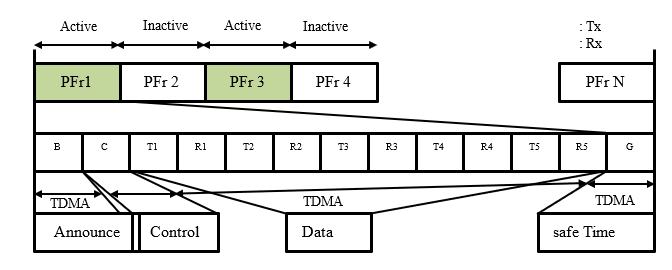

Supplement: S5 Fig — (ZIP) [file pone.0345290.s005.zip › S5_Fig.png]

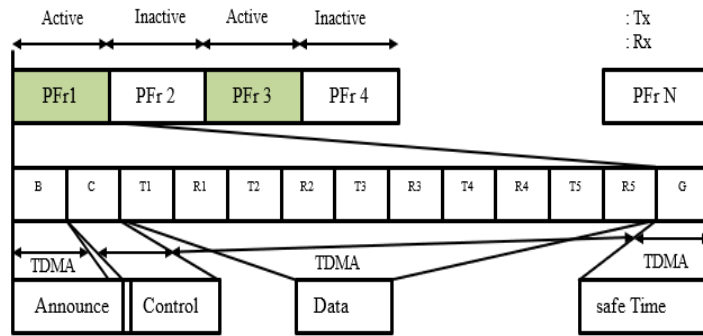

Figure 1: \*  
S5 Fig TDMA time-slot allocation in the proposed model

Supplement: S5 Fig — (ZIP) [file pone.0345290.s005.zip › S5_Fig.pdf]

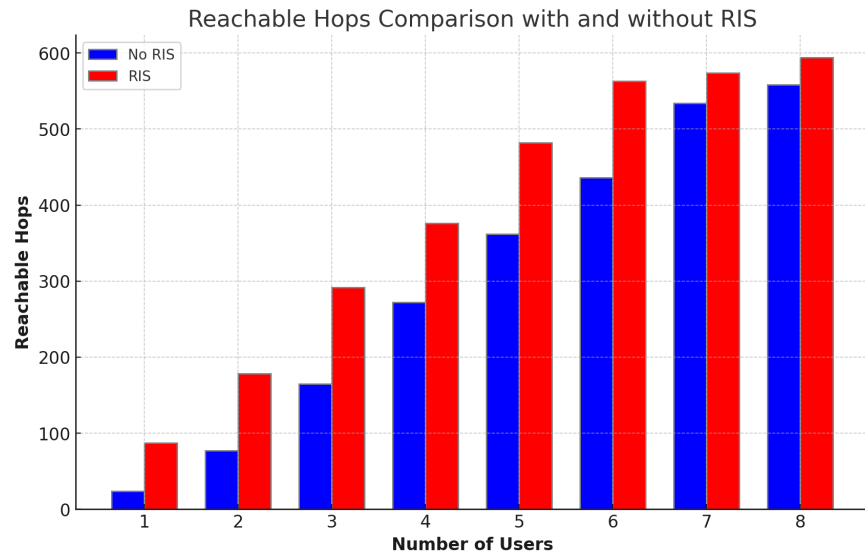

Figure 1: \*  
S6 Fig Reachable Hops Comparison With and Without RIS

Supplement: S6 Fig — (ZIP) [file pone.0345290.s006.zip › S6_Fig.pdf]

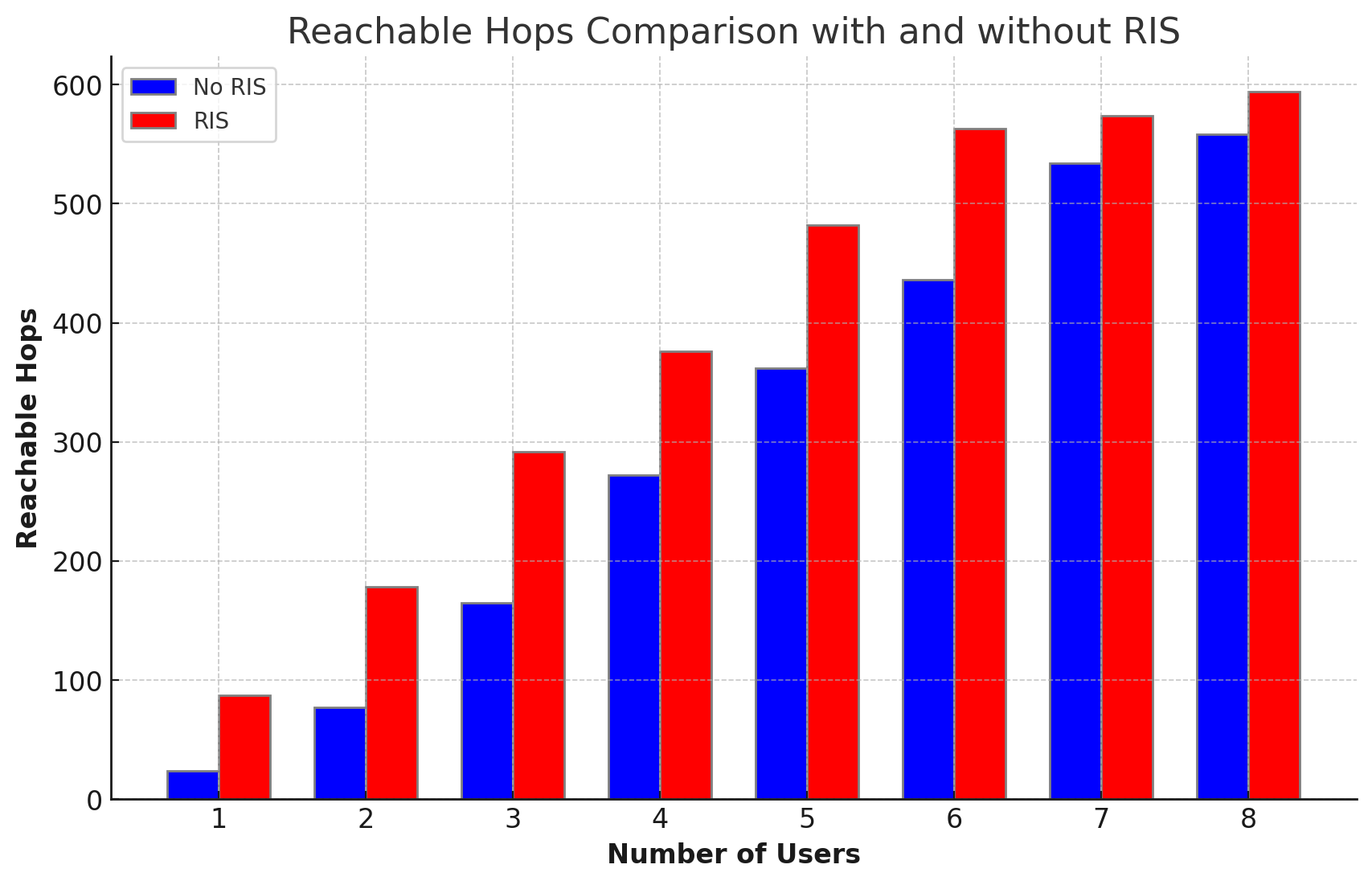

Supplement: S6 Fig — (ZIP) [file pone.0345290.s006.zip › S6_Fig.png]

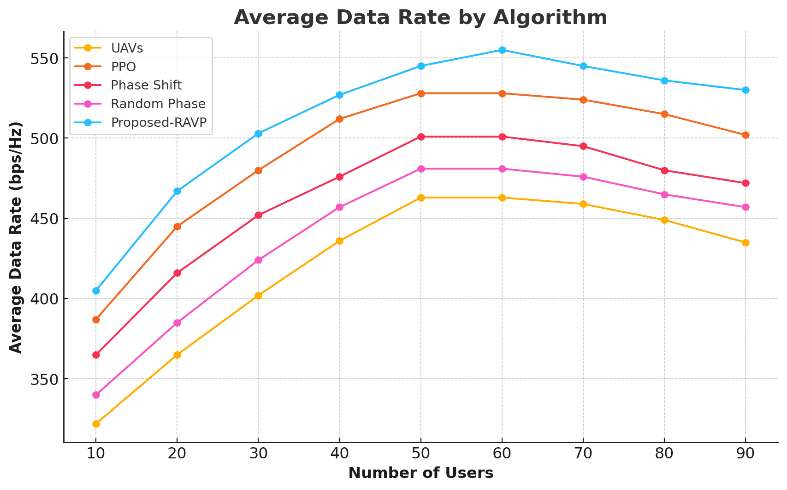

Supplement: S7 Fig — (ZIP) [file pone.0345290.s007.zip › S7_Fig.png]

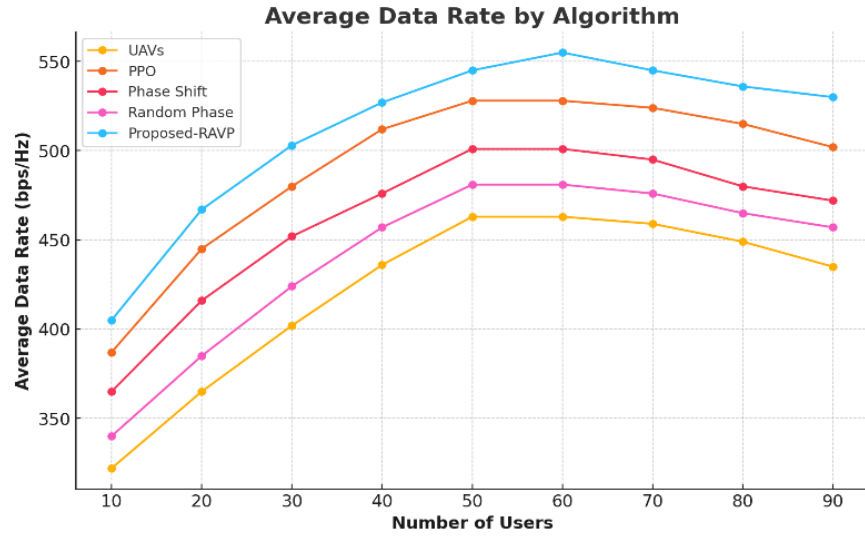

Figure 1: \*  
S7 Fig Impact of Number of Users on Average Data Rate (bps/Hz)

Supplement: S7 Fig — (ZIP) [file pone.0345290.s007.zip › S7_Fig.pdf]

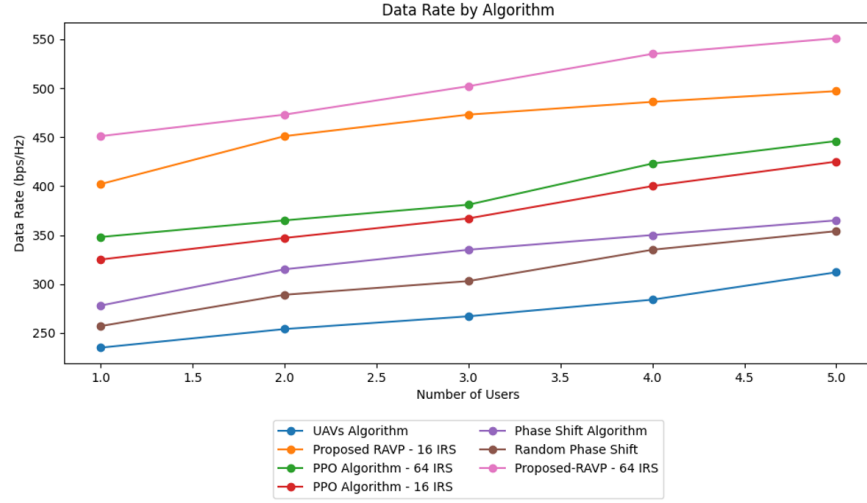

Figure 1: \*  
 S8 Fig Impact of Increasing the Number of Users on Data Rate (bps/Hz)

Supplement: S8 Fig — (ZIP) [file pone.0345290.s008.zip › S8_Fig.pdf]

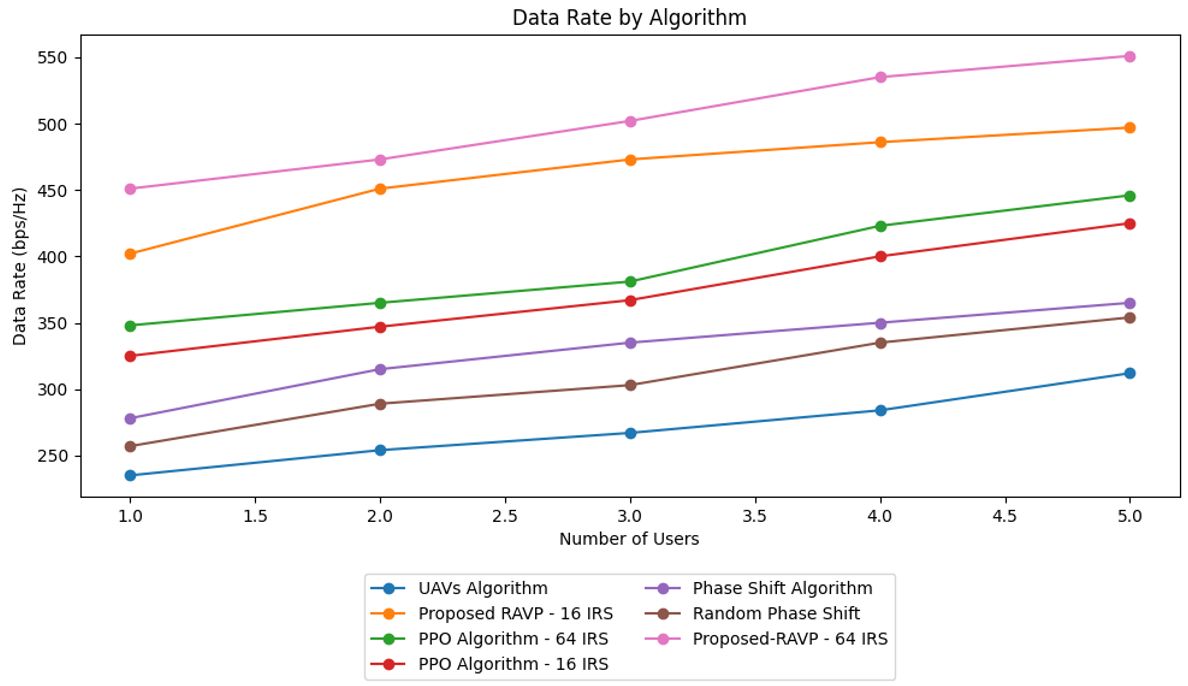

Supplement: S8 Fig — (ZIP) [file pone.0345290.s008.zip › S8_Fig.png]

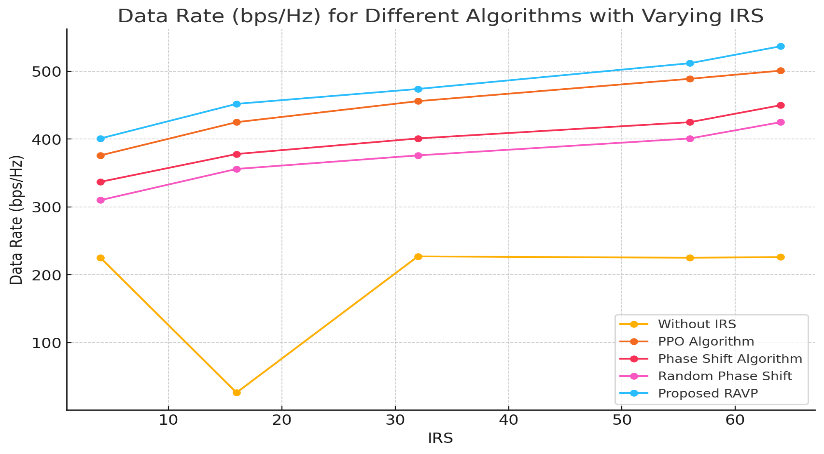

Supplement: S9 Fig — (ZIP) [file pone.0345290.s009.zip › S9_Fig.png]

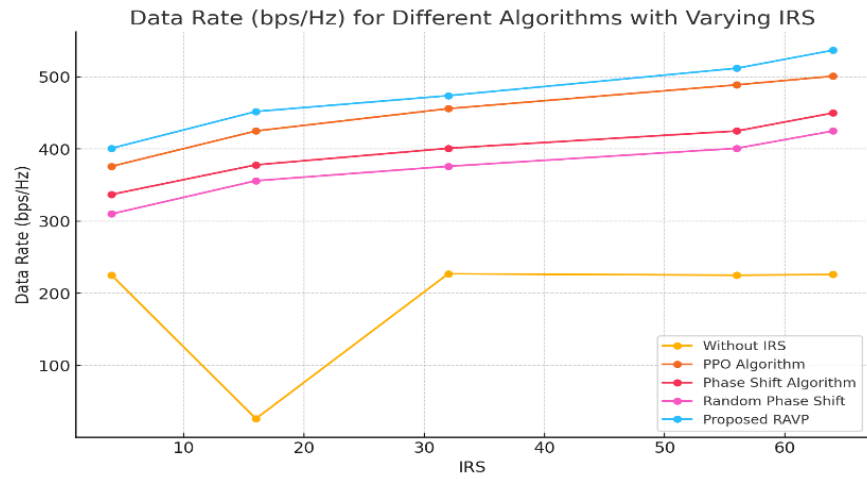

Figure 1: \*  
S9 Fig Impact of IRS on data rate (bps/Hz)

Supplement: S9 Fig — (ZIP) [file pone.0345290.s009.zip › S9_Fig.pdf]

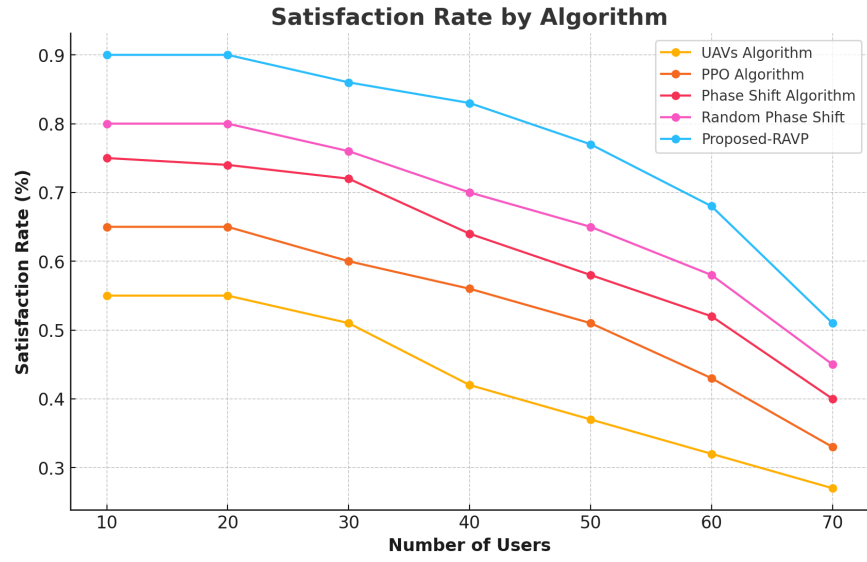

Figure 1: \*  
S10 Fig Impact of Number of Users on Satisfaction Rate (%)

Supplement: S10 Fig — (ZIP) [file pone.0345290.s010.zip › S10_Fig.pdf]

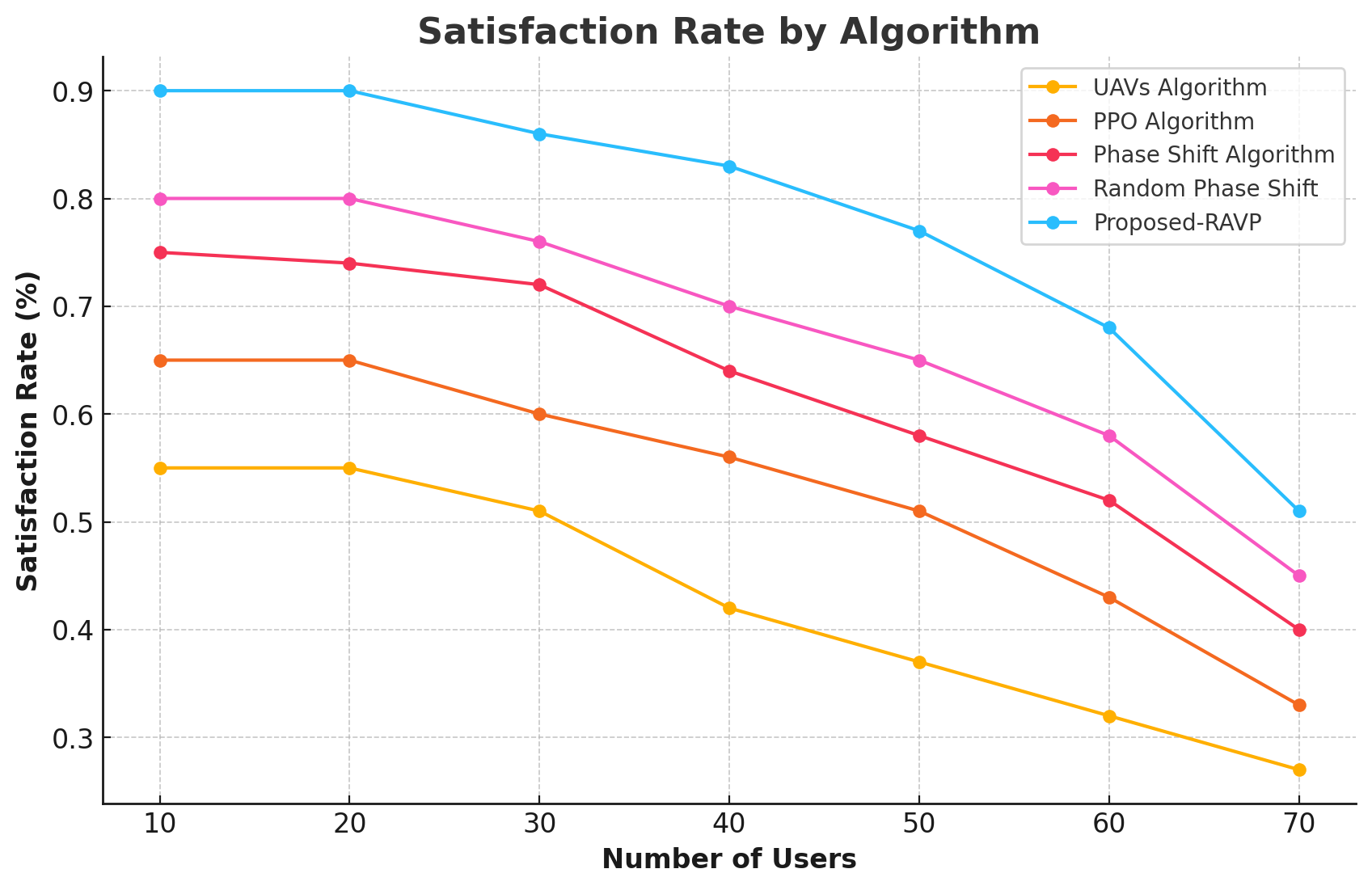

Supplement: S10 Fig — (ZIP) [file pone.0345290.s010.zip › S10_Fig.png]

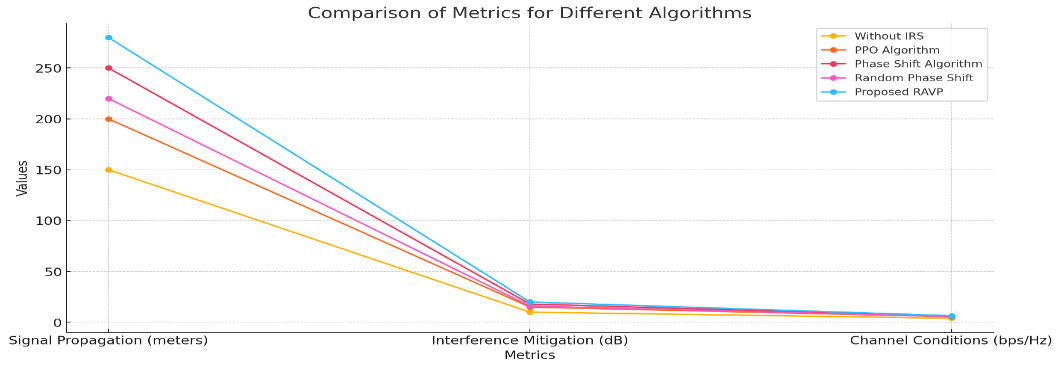

Supplement: S11 Fig — (ZIP) [file pone.0345290.s011.zip › S11_Fig.png]
