# Supplementary material for: Reconfigurable intelligent surface and UAV coordination for reliable THz wireless networks
Source: PLoS One. 2026 Mar 23;21(3):e0345290. doi: 10.1371/journal.pone.0345290 (PMC13008106; doi:10.1371/journal.pone.0345290)
Supplement: S11 Fig — (ZIP) [file pone.0345290.s011.zip › S11_Fig.pdf]

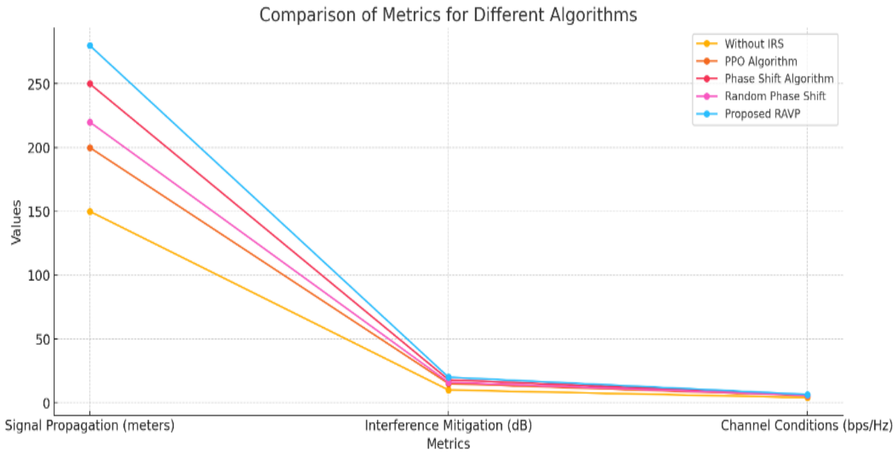

Figure 1: *
S11 Fig Impact of signal propagation, interference mitigation, and channel conditions (existing vs. proposed)
