# Supplementary material for: Reconfigurable intelligent surface and UAV coordination for reliable THz wireless networks
Source: PLoS One. 2026 Mar 23;21(3):e0345290. doi: 10.1371/journal.pone.0345290 (PMC13008106; doi:10.1371/journal.pone.0345290)
Supplement: S1 Table — (ZIP) [file pone.0345290.s012.zip › S1_Table.pdf]

Table 1: *
S1 Table Comparative analysis of UAV-enabled IoT and 6G communication frameworks

Work	Network Type	Key Technologies	Optimization / Intelligence	Target Applications	Limitations	Performance Focus
[?, ?]	IoT-UAV	IoT sensors, UAV platforms	Rule-based data aggregation	Environmental monitoring, agriculture	Limited adaptability, no learning	Data collection efficiency
[?, ?]	5G NR-UAV	UAV-assisted 5G NR	Experimental deployment	IoT connectivity, coverage extension	Fixed configurations	Connectivity reliability
[?, ?]	6G-UAV	3D beamforming, AI, DAE	Unsupervised learning	Secure UAV communications	Focus limited to secrecy	Secrecy rate improvement
[?, ?, ?, ?]	UAV-RIS	RIS, VLC, UAVs	Energy-aware optimization	Energy-efficient networking	Partial joint optimization	Energy utilization
[?]	IoT-RIS-UAV-THz	RIS, UAVs, NOMA, CPN	Centralized resource optimization	IoT computing and communication	Ideal CSI assumption, centralized control	Spectral efficiency, task reliability
[?]	RIS-THz	RIS, THz channel modeling	Analytical optimization	Static THz communication	No UAV mobility or learning	Coverage and link reliability
[?]	IoT-RIS-UAV-THz	RIS, UAVs, NOMA, CPN	Learning-based iterative optimization	Low-latency IoT services	High computational overhead	Throughput and latency
[?]	UAV-THz	Cooperative UAVs, energy-harvesting RIS	Energy-aware cooperative design	Energy-efficient THz networks	Hardware complexity	Energy efficiency, network lifetime
[?, ?]	6G NTN-UAIS	NTN, UAVs, autonomous systems	Distributed intelligence	Precision agriculture, disaster response	High system complexity	Autonomous coordination
Proposed Work	6G IoT-UAV-RIS-THz	UAVs, RIS, THz/mmWave	RL-based joint optimization	Reliable 6G IoT communication	UAV energy and hardware limits	Coverage, data rate, reliability
