# Supplementary material for: Reconfigurable intelligent surface and UAV coordination for reliable THz wireless networks
Source: PLoS One. 2026 Mar 23;21(3):e0345290. doi: 10.1371/journal.pone.0345290 (PMC13008106; doi:10.1371/journal.pone.0345290)
Supplement: S2 Table — (ZIP) [file pone.0345290.s013.zip › S2_Table.pdf]

Table 1: *
S2 Table Comprehensive simulation parameters

Simulation parameter	Value
Routing/Optimization Framework	RAVP
Number of IoT Users/Nodes	101
Simulation Area (X × Y)	1000×500 m^2
Simulation Time	200 units
Number of UAVs	Variable (scenario-dependent)
UAV Altitude	80-120 m
UAV Mobility Model	Controlled adaptive repositioning
Carrier Frequency (THz band)	0.3-1 THz
THz Molecular Absorption Coefficient $k(f)$	Frequency-dependent (standard THz model)
Noise Variance σ^2	10^{-9} W
Transmitter Power P_t	20 dBm
Path Loss Model	THz free-space with absorption loss
RIS Deployment Type	UAV-mounted / environment-assisted
RIS Geometry	Uniform planar array
Number of RIS Elements	4, 16, 32, 56, 64
RIS Element Spacing	$\lambda/2$
RIS Phase Resolution	Continuous
Propagation Environment	Obstacle-aware urban setting
Random Seed Values	Multiple fixed seeds
Monte Carlo Simulation Runs	100 independent trials
Performance Averaging Method	Mean over Monte Carlo runs
Reported Statistical Measure	Mean ± Standard Deviation
Confidence Level	95% (where applicable)
