# Supplementary material for: Reconfigurable intelligent surface and UAV coordination for reliable THz wireless networks
Source: PLoS One. 2026 Mar 23;21(3):e0345290. doi: 10.1371/journal.pone.0345290 (PMC13008106; doi:10.1371/journal.pone.0345290)
Supplement: S2a Table — (ZIP) [file pone.0345290.s014.zip › S2a_Table.pdf]

Table 1: *
S2a Table Reproducibility and statistical robustness reporting

**Reproducibility**	**Statistical robustness**
Channel coefficients	THz free-space spreading loss with molecular absorption; Rician fading for UAV/RIS-user links
RIS geometry	Uniform planar array; element spacing $\lambda/2$; continuous phase shifts; element counts $4, 16, 32, 56, 64$ (S2 Table).
Propagation assumptions	Obstacle-aware urban setting; direct BS-user links blocked; UAV/RIS-assisted link used (S2 Table and System Model).
Monte Carlo settings	$N = 100$ independent trials per reported point; results averaged over trials (S2 Table).
Random seeds	Multiple fixed and documented seeds used for reproducibility (S2 Table).
Confidence intervals	95% CI reported for key metrics using $\bar{x} \pm 1.96\, s/\sqrt{N}$ with $N = 100$
RL statistical robustness	PPO and proposed RAVP additionally averaged over multiple independent training runs (different seeds) with run-to-run variability reported
