# Supplementary material for: Reconfigurable intelligent surface and UAV coordination for reliable THz wireless networks
Source: PLoS One. 2026 Mar 23;21(3):e0345290. doi: 10.1371/journal.pone.0345290 (PMC13008106; doi:10.1371/journal.pone.0345290)
Supplement: S3 Table — (ZIP) [file pone.0345290.s015.zip › S3_Table.pdf]

Table 1: *
S3 Table Reachable hops with and without RIS

**Number of Users**	**No RIS**	**RIS**
1	24	87
2	77	178
3	165	292
4	272	376
5	362	482
6	436	563
7	534	574
8	558	594
