# Supplementary material for: Reconfigurable intelligent surface and UAV coordination for reliable THz wireless networks
Source: PLoS One. 2026 Mar 23;21(3):e0345290. doi: 10.1371/journal.pone.0345290 (PMC13008106; doi:10.1371/journal.pone.0345290)
Supplement: S4 Table — (ZIP) [file pone.0345290.s016.zip › S4_Table.pdf]

Table 1: *
S4 Table Average Data Rate of Existing vs. Proposed Methods

Users	UAVs	PPO	Phase Shift	Random Phase	Proposed-RAVP
10	322	387	365	340	405
20	365	445	416	385	467
30	402	480	452	424	503
40	436	512	476	457	527
50	463	528	501	481	545
60	463	528	501	481	555
70	459	524	495	476	545
80	449	515	480	465	536
90	435	502	472	457	530
