# Supplementary material for: Reconfigurable intelligent surface and UAV coordination for reliable THz wireless networks
Source: PLoS One. 2026 Mar 23;21(3):e0345290. doi: 10.1371/journal.pone.0345290 (PMC13008106; doi:10.1371/journal.pone.0345290)
Supplement: S4a Table — (ZIP) [file pone.0345290.s017.zip › S4a_Table.pdf]

Table 1: *
S4a Table Key results with statistical reporting (based on simulation parameters in S2 Table, $N = 100$ Monte Carlo trials)

**Metric (scenario)**	**Baseline**	**Proposed-RAVP**	**Reporting format**
Reachable hops (8 users; S3 Table)	558 (No RIS)	594 (RIS)	Mean; report as mean ± SD and 95% CI over $N = 100$
Average data rate (60 users; S4 Table)	528 (PPO)	555	Mean; report as mean ± SD and 95% CI over $N = 100$
Data rate vs. IRS (64 elements; S6 Table)	501 (PPO)	537	Mean; report as mean ± SD and 95% CI over $N = 100$
Satisfaction rate (10 users; S7 Table)	0.80 (Random phase)	0.90	Mean; report as mean ± SD and 95% CI over $N = 100$
Signal propagation (S8 Table)	150 (Without IRS)	280	Mean; report as mean ± SD and 95% CI over $N = 100$
Interference mitigation (S8 Table)	10 (Without IRS)	20	Mean; report as mean ± SD and 95% CI over $N = 100$
Channel conditions (S8 Table)	4.0 (Without IRS)	6.5	Mean; report as mean ± SD and 95% CI over $N = 100$
