# Supplementary material for: Reconfigurable intelligent surface and UAV coordination for reliable THz wireless networks
Source: PLoS One. 2026 Mar 23;21(3):e0345290. doi: 10.1371/journal.pone.0345290 (PMC13008106; doi:10.1371/journal.pone.0345290)
Supplement: S5 Table — (ZIP) [file pone.0345290.s018.zip › S5_Table.pdf]

Table 1: *
S5 Table Data Rate of Existing vs. Proposed Methods (bps/Hz)

Users	UAV algorithm	Proposed RAVP - 16 IRS	PPO Algorithm - 64 IRS	PPO Algorithm - 16 IRS	Phase Shift Algorithm	Random Phase Shift	Proposed-RAVP - 64 IRS
1	235	402	348	325	278	257	451
2	254	451	365	347	315	289	473
3	267	473	381	367	335	303	502
4	284	486	423	400	350	335	535
5	312	497	446	425	365	354	551
