# Supplementary material for: Reconfigurable intelligent surface and UAV coordination for reliable THz wireless networks
Source: PLoS One. 2026 Mar 23;21(3):e0345290. doi: 10.1371/journal.pone.0345290 (PMC13008106; doi:10.1371/journal.pone.0345290)
Supplement: S6 Table — (ZIP) [file pone.0345290.s019.zip › S6_Table.pdf]

Table 1: *
S6 Table Data Rate With and Without IRS (bps/Hz)

| IRS | Without IRS | PPO Algorithm | Phase Shift Algorithm | Random Phase Shift | Proposed-RAVP |
|---|---|---|---|---|---|
| 4 | 225 | 376 | 337 | 310 | 401 |
| 16 | 226 | 425 | 378 | 356 | 452 |
| 32 | 227 | 456 | 401 | 376 | 474 |
| 56 | 225 | 489 | 425 | 401 | 512 |
| 64 | 226 | 501 | 450 | 425 | 537 |
