# Supplementary material for: Reconfigurable intelligent surface and UAV coordination for reliable THz wireless networks
Source: PLoS One. 2026 Mar 23;21(3):e0345290. doi: 10.1371/journal.pone.0345290 (PMC13008106; doi:10.1371/journal.pone.0345290)
Supplement: S7 Table — (ZIP) [file pone.0345290.s020.zip › S7_Table.pdf]

Table 1: *
S7 Table Satisfaction Rate of Existing vs. Proposed Methods

Users	UAVs Algorithm	PPO Algorithm	Phase Shift Algorithm	Random Phase Shift	Proposed-RAVP
10	0.55	0.65	0.75	0.80	0.90
20	0.55	0.65	0.74	0.80	0.90
30	0.51	0.60	0.72	0.76	0.86
40	0.42	0.56	0.64	0.70	0.83
50	0.37	0.51	0.58	0.65	0.77
60	0.32	0.43	0.52	0.58	0.68
70	0.27	0.33	0.40	0.45	0.51
