# Supplementary material for: Reconfigurable intelligent surface and UAV coordination for reliable THz wireless networks
Source: PLoS One. 2026 Mar 23;21(3):e0345290. doi: 10.1371/journal.pone.0345290 (PMC13008106; doi:10.1371/journal.pone.0345290)
Supplement: S8 Table — (ZIP) [file pone.0345290.s021.zip › S8_Table.pdf]

Table 1: *
S8 Table Comparison of signal propagation, interference mitigation, and optimized channel conditions

Metric	Without IRS	PPO Algorithm	Phase Shift Algorithm	Random Phase Shift	Proposed-RAVP
**Signal Propagation (m)**	150	200	250	220	280
**Interference Mitigation (dB)**	10	15	18	16	20
**Channel Conditions (bps/Hz)**	4.0	5.5	6.0	5.7	6.5
