# Supplementary material for: Reconfigurable intelligent surface and UAV coordination for reliable THz wireless networks
Source: PLoS One. 2026 Mar 23;21(3):e0345290. doi: 10.1371/journal.pone.0345290 (PMC13008106; doi:10.1371/journal.pone.0345290)
Supplement: S9 Table — (ZIP) [file pone.0345290.s022.zip › S9_Table.pdf]

Table 1: *

S9 Table Comparative analysis of state-of-the-art methods against the proposed RAVP framework using aligned performance metrics

| **Method** | **Reachable Hops** | **Average Data Rate** | **Signal Propagation** | **Interference Mitigation** | **Channel Conditions** | **Adaptability/Scalability** |
|---|---|---|---|---|---|---|
| Du *et al.* (2022) [?] | Not reported | Moderate | Moderate | Moderate | Moderate | Low |
| Pan *et al.* (2025) [?] | Not reported | Moderate–High | Moderate | Moderate | Moderate | Moderate (static optimization) |
| Pan *et al.* (2025) [?] | Not reported | High | Moderate | Moderate | Moderate | Moderate–High (iterative learning) |
| Song *et al.* (2025) [?] | Not reported | Moderate | High | Moderate | High | Moderate (hardware complexity) |
| Proposed RAVP | High | Highest | Highest | Highest | Highest | High (RL-based joint optimization) |
