# Supplementary material for: Reconfigurable intelligent surface and UAV coordination for reliable THz wireless networks
Source: PLoS One. 2026 Mar 23;21(3):e0345290. doi: 10.1371/journal.pone.0345290 (PMC13008106; doi:10.1371/journal.pone.0345290)
Supplement: S9a Table — (ZIP) [file pone.0345290.s023.zip › S9a_Table.pdf]

Table 1: *
S9a Table Quantitative metric comparison with recent state-of-the-art methods [?, ?, ?, ?]

**Method**	**Throughput (bps/Hz)**	**E2E latency (ms)**	**Reliability**	**Energy eff. (bits/J)**	**ΔThroughput vs. best SOTA**	**ΔLatency vs. best SOTA**	**ΔReliability vs. best SOTA**
Du *et al.* (2022) [?]	505	20.0	0.91	4.6	-	-	-
Pan *et al.* (2025) [?]	515	19.5	0.92	4.7	-	-	-
Pan *et al.* (2025) [?]	520	19.0	0.93	4.8	-	-	-
Song *et al.* (2025) [?]	525	18.5	0.94	4.9	-	-	-
Proposed RAVP	555	15.0	0.96	5.4	+5.7%	−18.9%	+2.1%
