# Supplementary material for: Reconfigurable intelligent surface and UAV coordination for reliable THz wireless networks
Source: PLoS One. 2026 Mar 23;21(3):e0345290. doi: 10.1371/journal.pone.0345290 (PMC13008106; doi:10.1371/journal.pone.0345290)
Supplement: S9b Table — (ZIP) [file pone.0345290.s024.zip › S9b_Table.pdf]

Table 1: *
S9b Table Statistical reporting and robustness summary for S9a results ($N = 100$ Monte Carlo trials; fixed seeds; RL multi-run variability)

**Method**	**Throughput (bps/Hz)**	**E2E latency (ms)**	**Reliability**	**Energy eff. (bits/J)**
Du *et al.* (2022) [?]	505 ± 11 (95% CI: [503, 507])	20.0 ± 0.9 (95% CI: [19.8, 20.2])	0.91 ± 0.01 (95% CI: [0.908, 0.912])	4.6 ± 0.2 (95% CI: [4.56, 4.64])
Pan *et al.* (2025) [?]	515 ± 10 (95% CI: [513, 517])	19.5 ± 0.8 (95% CI: [19.3, 19.7])	0.92 ± 0.01 (95% CI: [0.918, 0.922])	4.7 ± 0.2 (95% CI: [4.66, 4.74])
Pan *et al.* (2025) [?]	520 ± 9 (95% CI: [518, 522])	19.0 ± 0.8 (95% CI: [18.8, 19.2])	0.93 ± 0.01 (95% CI: [0.928, 0.932])	4.8 ± 0.2 (95% CI: [4.76, 4.84])
Song *et al.* (2025) [?]	525 ± 9 (95% CI: [523, 527])	18.5 ± 0.7 (95% CI: [18.3, 18.7])	0.94 ± 0.01 (95% CI: [0.938, 0.942])	4.9 ± 0.2 (95% CI: [4.86, 4.94])
Proposed RAVP	555 ± 8 (95% CI: [553, 557])	15.0 ± 0.6 (95% CI: [14.9, 15.1])	0.96 ± 0.01 (95% CI: [0.958, 0.962])	5.4 ± 0.2 (95% CI: [5.36, 5.44])
RL training variability (RAVP; 5 runs)	Mean 555; run SD = 6	Mean 15.0; run SD = 0.4	Mean 0.96; run SD = 0.003	Mean 5.4; run SD = 0.08
